# Supplementary material for: VAMP4 Is an Essential Cargo Molecule for Activity-Dependent Bulk Endocytosis
Source: Neuron. 2015 Dec 2;88(5):973–84. doi: 10.1016/j.neuron.2015.10.043 (PMC4678114; doi:10.1016/j.neuron.2015.10.043)
Supplement: Document S1. Figures S1–S8 [file mmc1.pdf]

Neuron

Supplemental Information

VAMP4 Is an Essential Cargo Molecule for Activity-Dependent Bulk Endocytosis

Jessica C. Nicholson-Fish, Alexandros C. Kokotos, Thomas H. Gillingwater, Karen J. Smillie, and Michael A. Cousin

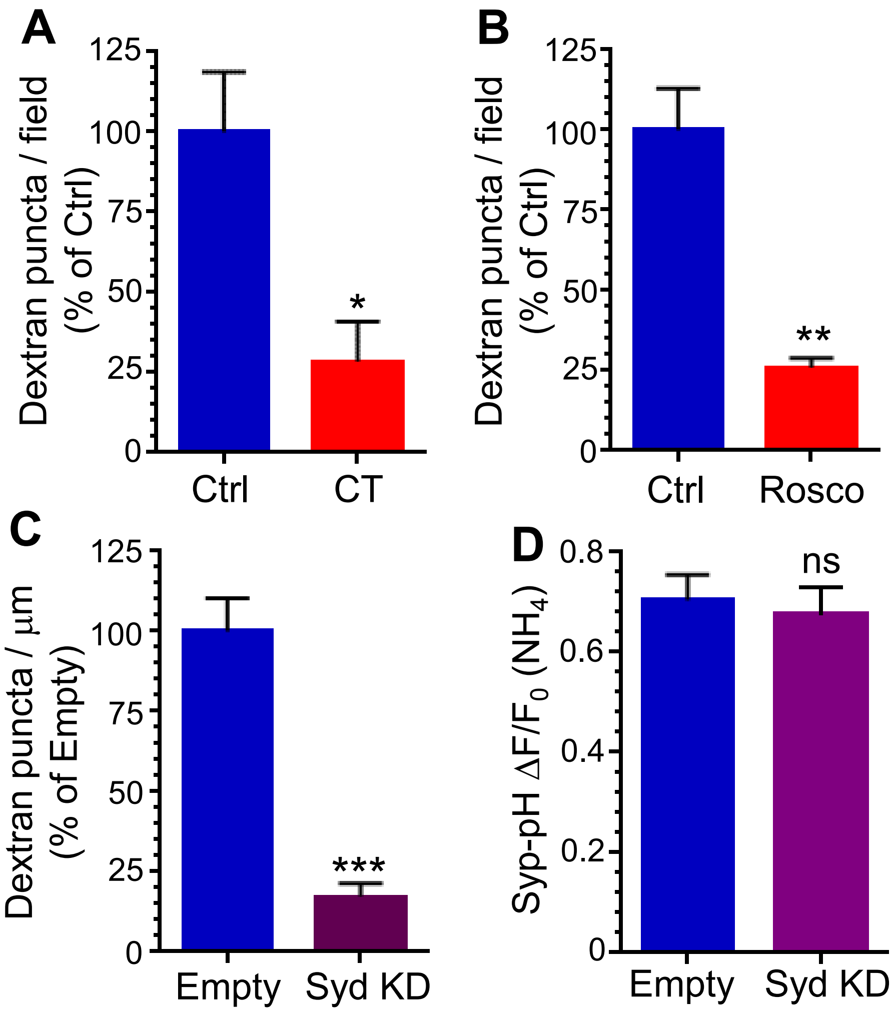

Figure S1 – *Related to Figure 2: Inhibition of ADBE arrests TMR-dextran uptake.* **A,B**) Cerebellar neurons were stimulated with two sequential action potential trains (S1 and S2) 10 minutes apart (both 40 Hz 10 s). Cultures were incubated with either **A)** 2 µM CT99021 (CT) or **B)** 10 µM roscovitine (Ros) 10 minutes before S1. At S2 50 µM TMR-dextran was added and immediately washed away on termination of stimulation. Quantification of dextran puncta per field ± SEM normalised to control is displayed (n = 4 Ctrl, n = 5 CT, n = 3 for Ctrl and Ros, ** = p<0.01, * = p<0.05, students t test). **C**) Cerebellar neurons were transfected with either empty shRNA vector or shRNA against syndapin I (Syd KD). Cultures were stimulated at 40 Hz for 10 s in the presence of 50 µM TMR-dextran. Quantification of dextran puncta per µm ± SEM normalised to empty control (n = 33 Empty, n = 40 Syd KD, *** = p<0.01, students t test). **D**) Cerebellar neurons transfected with synaptophysin-pHluorin (syp-pH) and either empty shRNA (empty) or shRNA against syndapin I (Syd KD) were stimulated with a train of 400 action potentials (40 Hz). Quantification of the evoked syp-pH response $\Delta F/F_0$ ± SEM normalised to the total pHluorin pool (NH_4) for both empty (blue) and Syd KD (purple), n = 8 Empty, n = 5 Syd KD, ns, students t test.

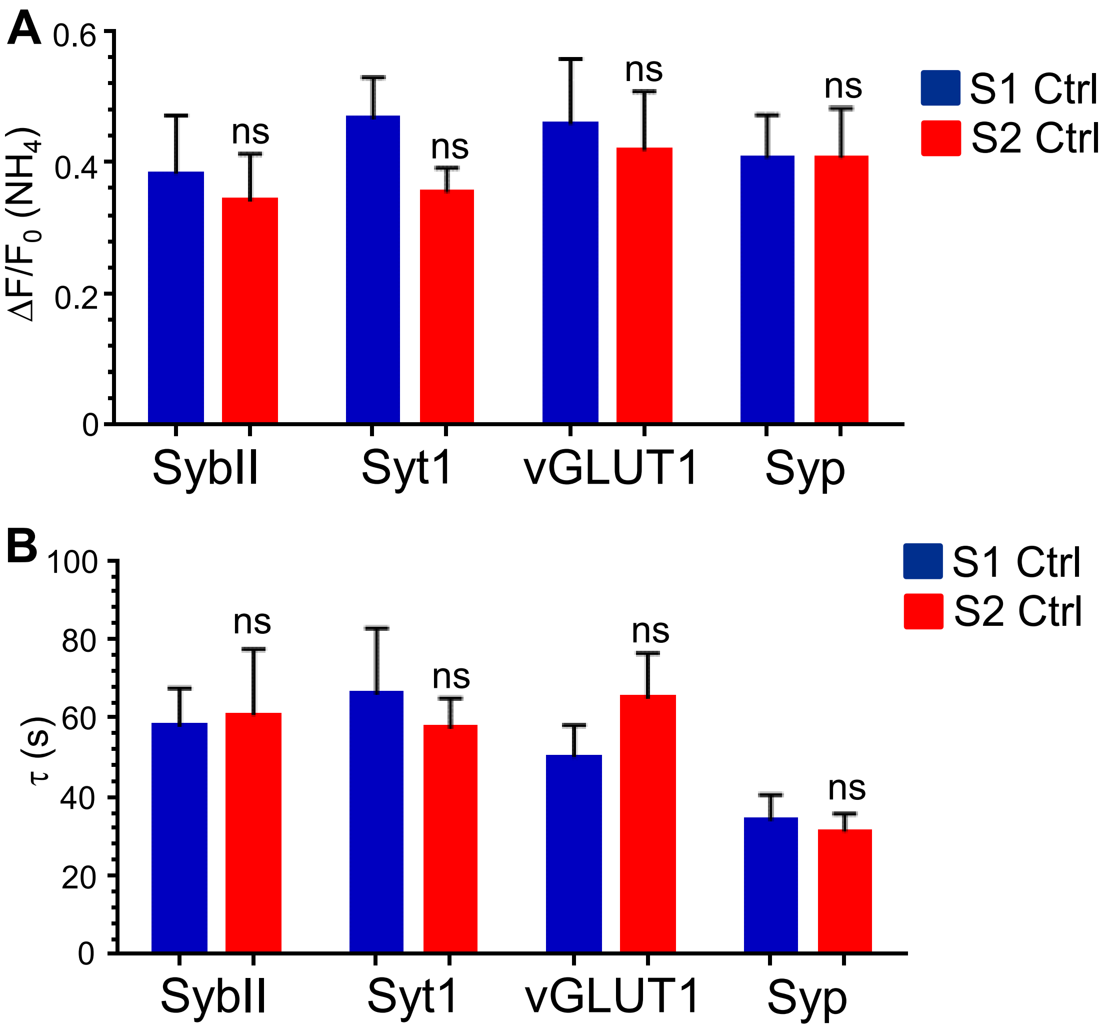

Figure S2 – *Related to Figure 4: Evoked response of multiple pHluorin reporters is reproducible during intense stimulation.* Cerebellar neurons transfected with the pHluorin reporters synaptobrevinII-pHluorin (SybII), synaptotagmin-1-pHluorin (Syt1), vGLUT1-pHluorin (vGLUT1) or synaptophysin-pHluorin (Syp) were stimulated with two sequential action potential trains (S1 and S2) 10 minutes apart (both 40 Hz 10 s). **A**) Quantification of the evoked pHluorin peak response ($\Delta F/F_0$ ± SEM) normalised to the total pHluorin pool (NH_4) for both S1 (blue) and S2 (red) traces. **B**) Quantification of the average time constant (τ) ± SEM for both S1 (blue) and S2 (red) traces (n = 4 SybII, n = 10 Syt1, n = 5 vGLUT1, n = 6 Syp, ns students t test).

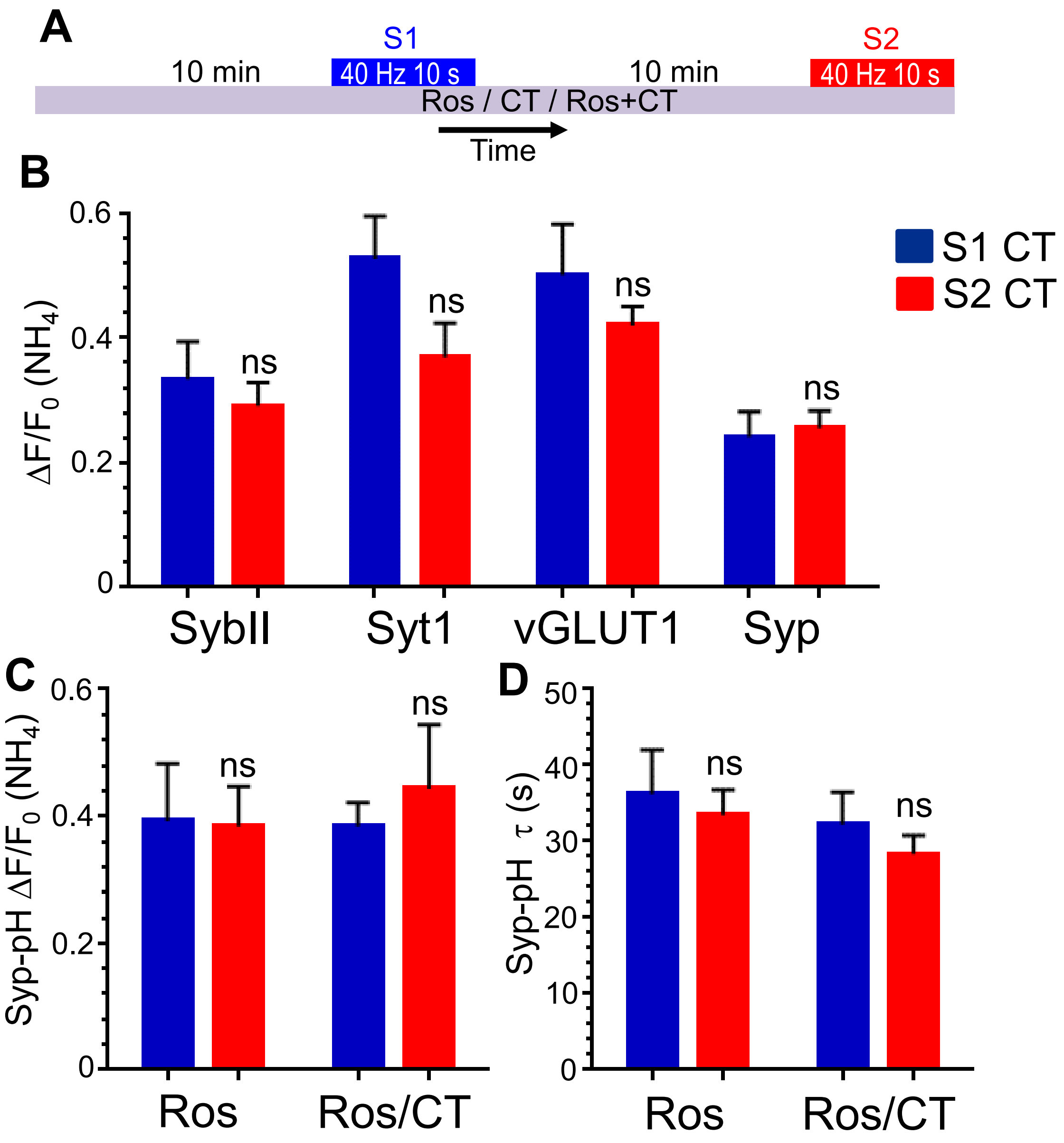

Figure S3 – *Related to Figure 4: Inhibition of ADBE does not affect the extent of response for multiple pHluorin reporters.* **A)** Cerebellar neurons transfected with the pHluorin reporters synaptobrevinII-pHluorin (SybII), synaptotagmin-1-pHluorin (Syt1), vGLUT1-pHluorin (vGLUT1) or synaptophysin-pHluorin (Syp) were stimulated with two sequential action potential trains (S1 and S2) 10 minutes apart (both 40 Hz 10 s). Cultures were incubated with either 2 µM CT99021 (CT), 10 µM roscovitine (Ros) or both 10 minutes prior to S1 and then continuously onwards. **B**) Quantification of the evoked pHluorin response $\Delta F/F_0$ ± SEM normalised to the total pHluorin pool (NH_4) for SybII, Syt1, vGLUT1 and Syp response for both S1 (blue) and S2 (red) traces in the presence of CT (n = 4 SybII, n = 10 Syt1, n = 5 vGLUT1, n = 6 Syp, ns students t test). **C**) Quantification of the evoked Syp-pH response $\Delta F/F_0$ ± SEM normalised to the total pHluorin pool (NH_4) or **D**) average time constant (τ) ± SEM for both S1 (blue) and S2 (red) in the presence of either Ros or both CT and Ros (n = 5 Ros, n = 4 Ros/CT, ns students t test)).

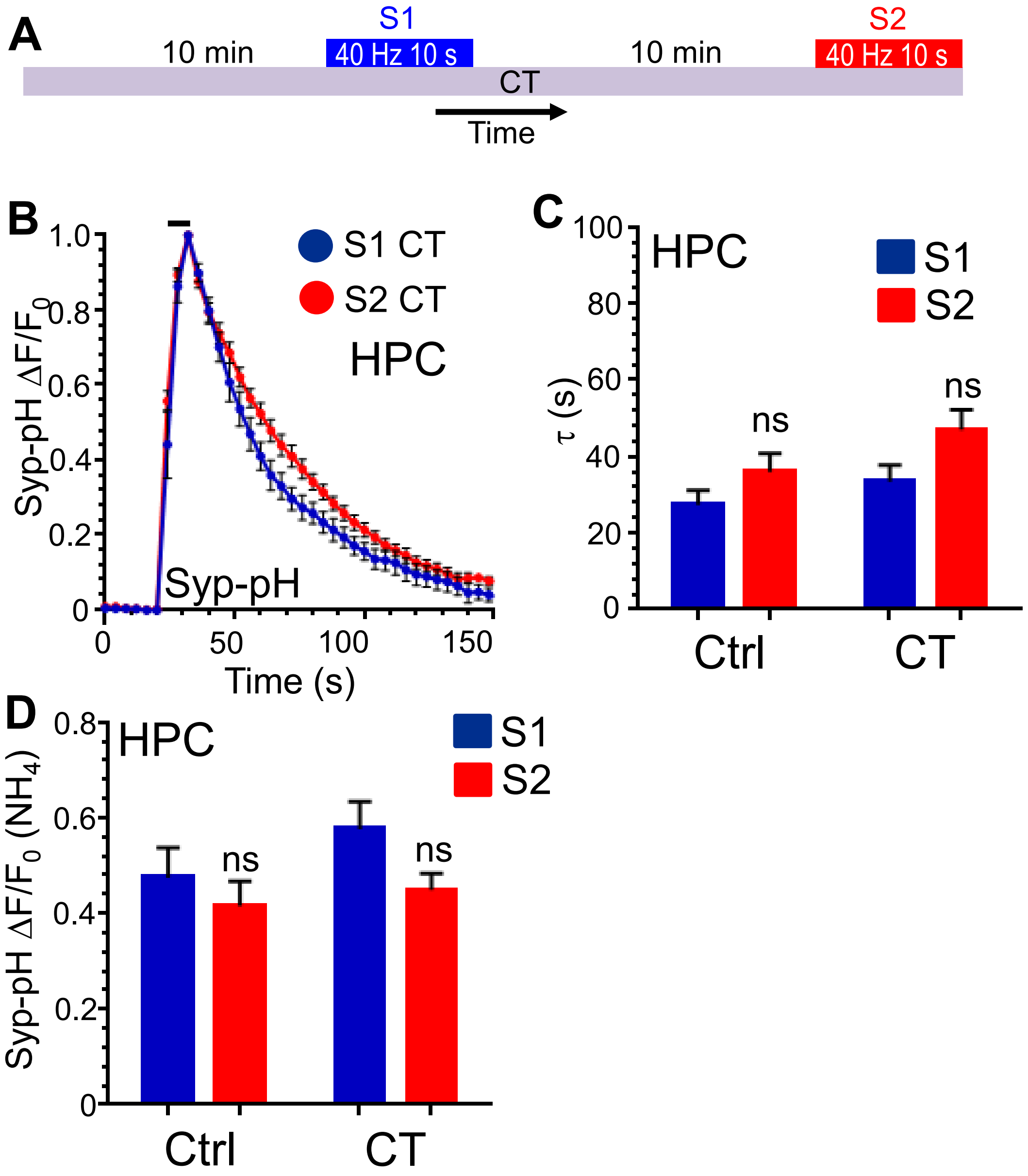

Figure S4 – *Related to results section paragraph 9: Synaptophysin-pHluorin is not retrieved by ADBE in hippocampal neurons.* **A**) Hippocampal (HPC) neurons transfected with synaptophysin-pHluorin (syp-pH) were stimulated with two sequential action potential trains (S1 and S2) 10 minutes apart (both 40 Hz 10 s). Cultures were incubated with 2 µM CT99021 (CT) 10 minutes prior to S1 and then continuously onwards where indicated. **B**) Average time course of the syp-pH fluorescent response in the presence of CT presented as $\Delta F/F_0$ ± SEM at both S1 (blue) and S2 (red). Bar indicates period of stimulation. **C**) Quantification of the average time constant (τ) ± SEM for both S1 (blue) and S2 (red). **D**) Quantification of the evoked syp-pH response $\Delta F/F_0$ ± SEM normalised to the total pHluorin pool (NH_4) for both S1 (blue) and S2 (red), n = 4 Ctrl, n = 5 CT, ns, one-way ANOVA.

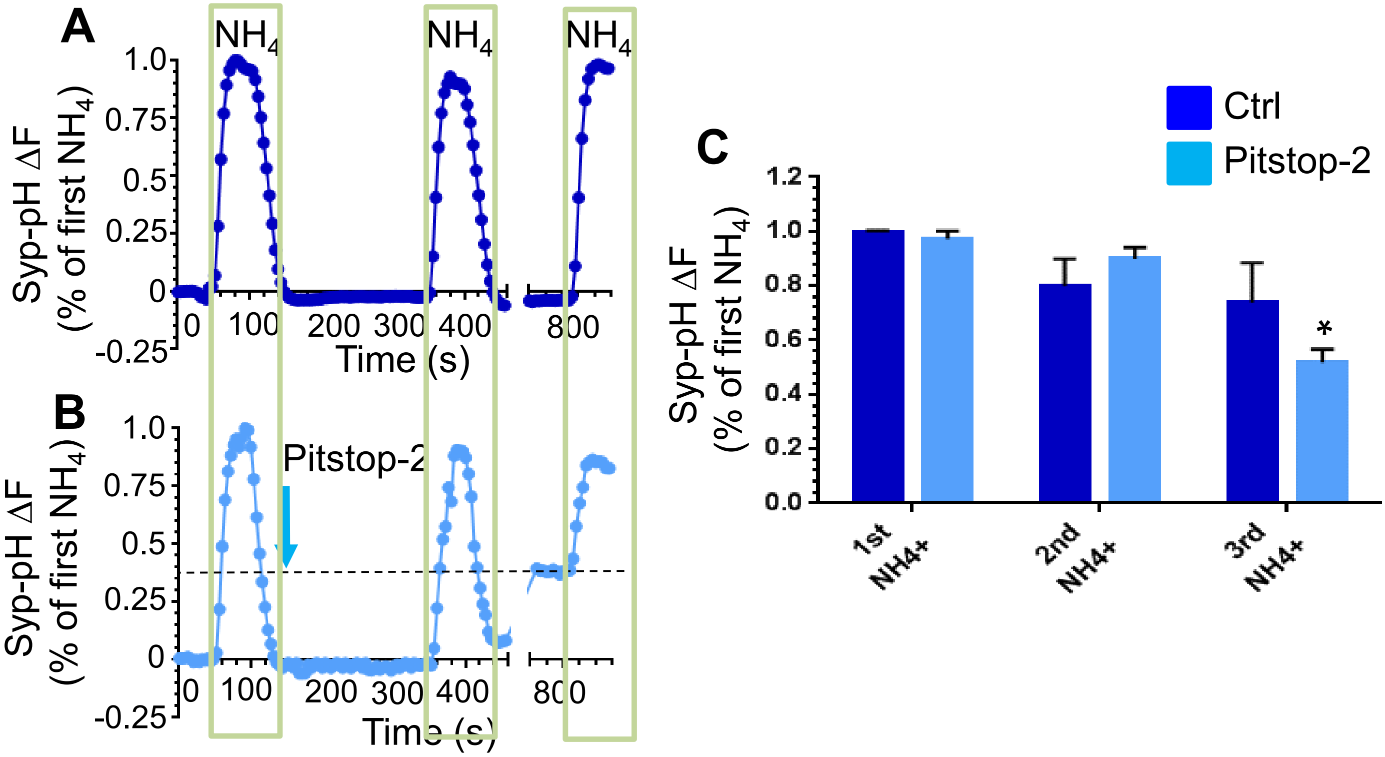

Figure S5 – *Related to Figure 5: Pitstop-2 only affects SV acidification after 10 minutes.* **A)** Cerebellar neurons transfected with synaptophysin-pHluorin (syp-pH) were challenged with a pulse of ammonium buffer (NH_4) to determine the maximum dynamic range of the fluorescent response (indicated by shaded area). Neurons were subsequently challenged with ammonium buffer after a further 4 minutes and 10 minutes. **B)** Neurons were subjected to an identical protocol apart from incubation with 15 µM pitstop-2 immediately after the first alkaline challenge (arrow). **C)** Dynamic range of the syp-pH response with (light blue) and without (dark blue) pitstop-2 is presented ± SEM (normalised to the first ammonia pulse, n = 3 for both, * = $p<0.05$, one-way ANOVA). Pitstop-2 reduces the syp-pH dynamic range after 10 mins, which is not within the time course of the experiments performed in this manuscript.

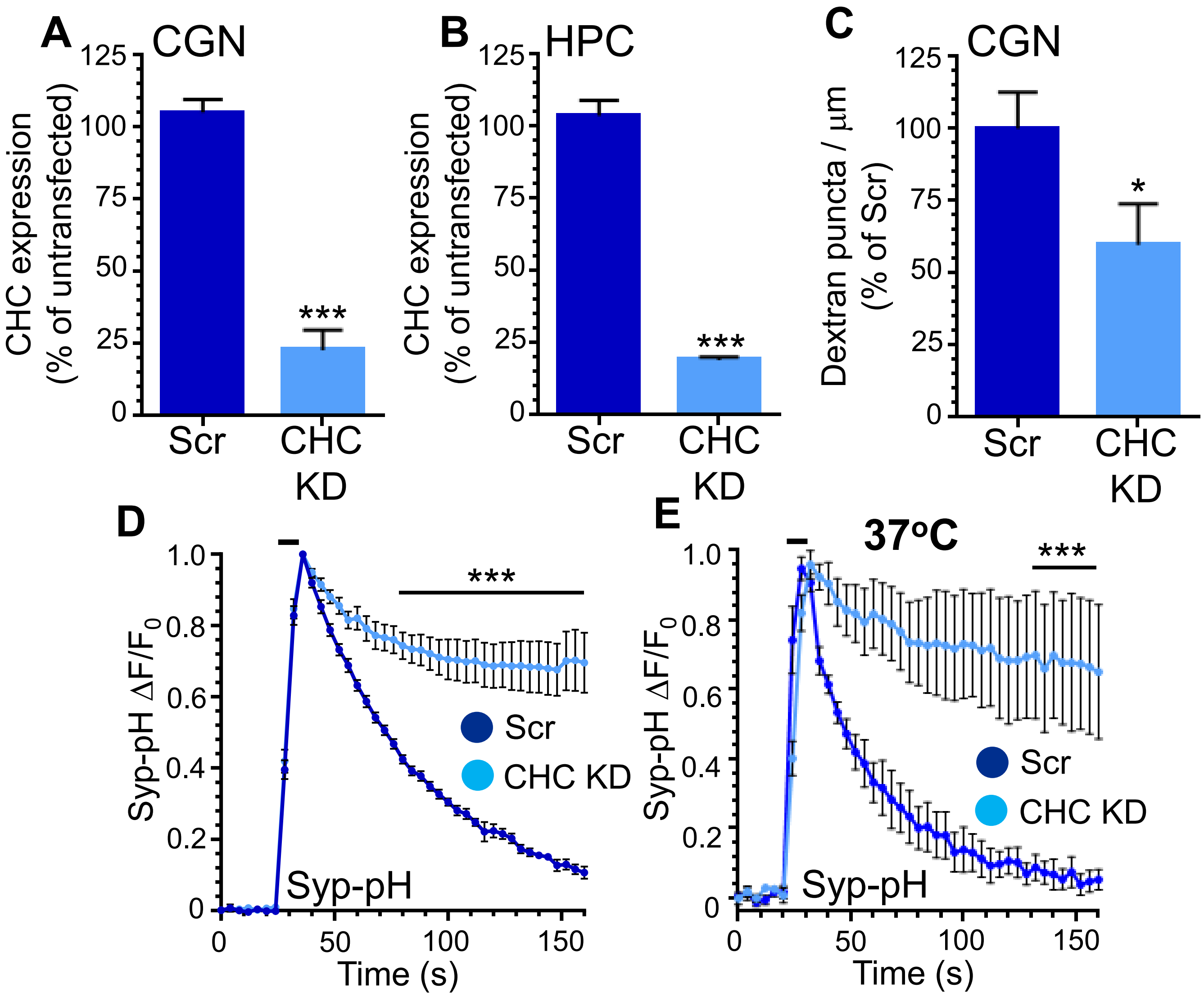

Figure S6 – *Related to Figure 5: Inhibition of CME arrests syp-pHluorin retrieval during intense stimulation.* **A,B)** Cerebellar (CGN) or hippocampal (HPC) neurons were transfected with either scrambled shRNA (Scr) or clathrin heavy chain (CHC) shRNA (CHC KD). The extent of CHC knockdown was quantified by immunofluorescence and presented as a percentage of untransfected neurons in the same field of view ± SEM (CGN, n=16 Scr, n=12 CHC KD, HPC, n=16 Scr, n=15, CHC KD, *** = p<0.001 students t test). **C)** CGNs transfected with either Scr or CHC KD shRNA were stimulated with an action potential train (40 Hz 10 s) in the presence of 50 mM TMR-dextran. Quantification of dextran puncta per µm ± SEM normalised to Scr control (n = 28 Scr, n = 30 CHC, * = p<0.05, students t test). **D,E)** CGNs or HPCs transfected with synaptophysin-pHluorin (syp-pH) and either Scr or CHC KD shRNA were stimulated (40 Hz 10 s) as indicated by bar. Average time course $\Delta F/F_0$ ± SEM of the evoked syp-pH response is displayed (CGN, n = 7 Scr, n = 8 CHC KD, HPC, n = 5 Scr, n = 4 CHC KD *** = p<0.001, two-way ANOVA). In **(E)** experiments were performed at physiological temperature (37°C).

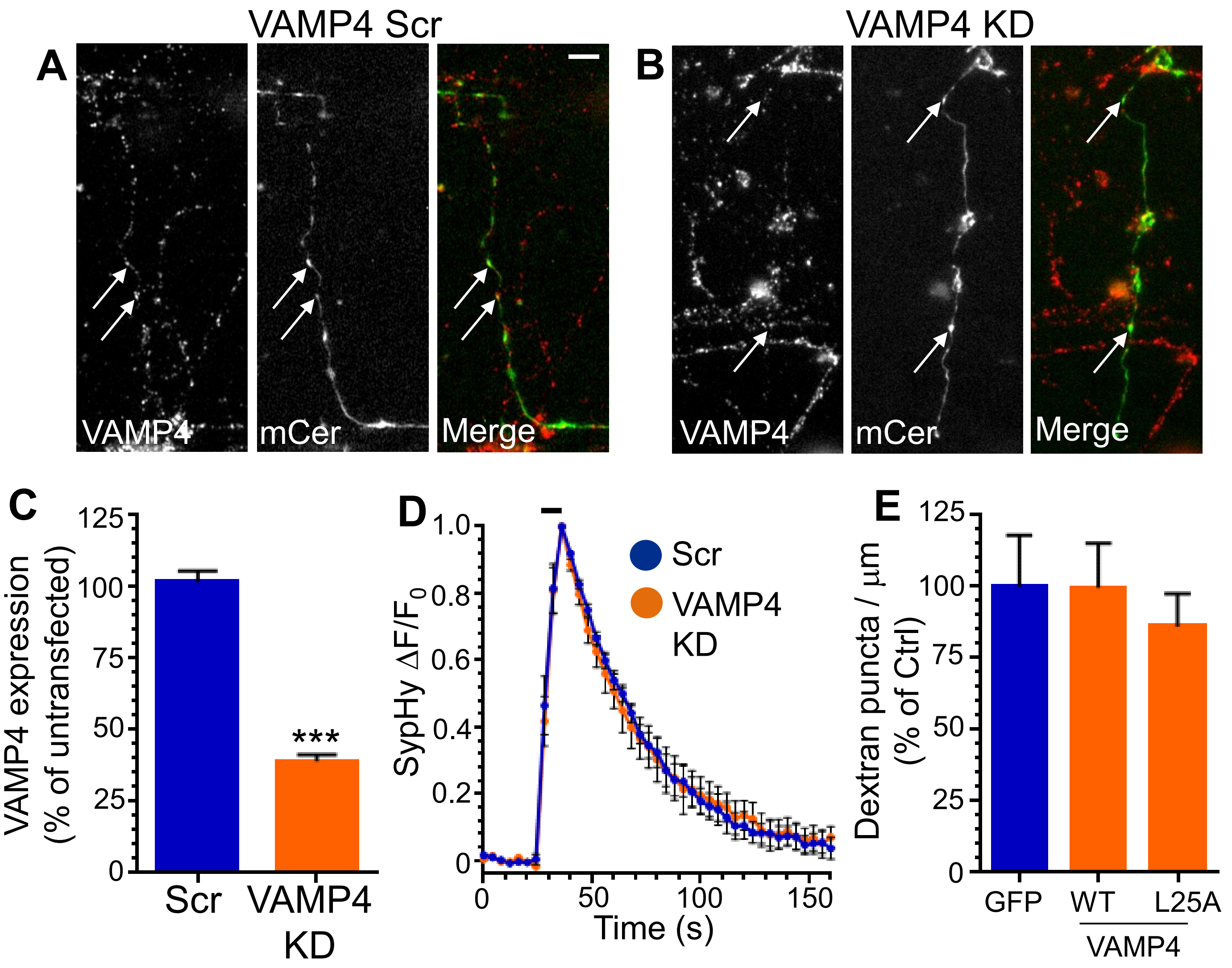

Supplementary Figure 7 – *Related to Figures 7 & 8: VAMP4 is essential for ADBE*. **A,B)** Immunofluorescence images show cerebellar neurons (CGNs) transfected with either scrambled (Scr, **A**) or VAMP4 shRNA (VAMP4 KD, **B**). Images display transfected neuron (green) VAMP4 expression (red) and a merged image. Arrows indicate example nerve terminals. Scale bar indicates 20 µm. **C)** Quantification of VAMP4 expression as a percentage of untransfected control (Scr, blue bars; VAMP4 KD, orange bars ± SEM (n = 4 coverslips for both, *** p<0.01, students t test). **D)** CGNs were transfected with synaptophysin-pHluorin (syp-pH) and either Scr or VAMP4 shRNA and stimulated with an action potential train (40 Hz 10 s). Bar indicates period of stimulation (n = 8 Scr, n = 6 VAMP4 KD, ns two-way-ANOVA). **E)** CGNs were transfected with either GFP, wild-type or L25A VAMP4-pHluorin and stimulated with 400 action potentials (40 Hz) in the presence of 50 µM TMR-dextran. Quantification of TMR-dextran puncta per µm ± SEM normalised to GFP control ± SEM (n = 23 GFP, n = 24 VAMP4 WT, n = 25 VAMP4 L25A, ns, one-way ANOVA).

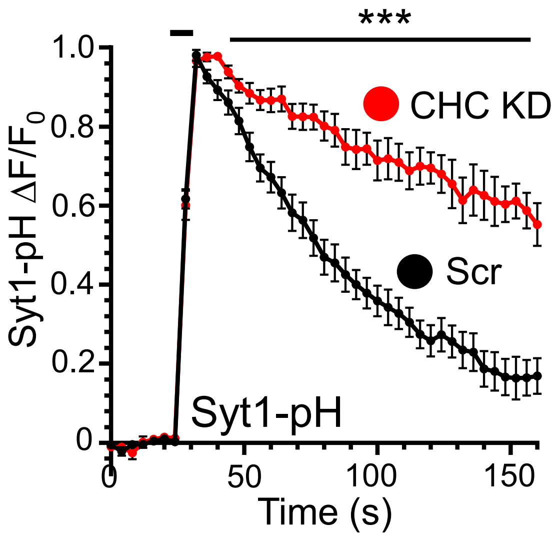

Supplementary Figure 8 – *Related to discussion section paragraph 2: Synaptotagmin-1-pHluorin retrieval is retarded by CHC shRNA.* Hippocampal neurons transfected with synaptotagmin-1-pHluorin (Syt1-pH) and either Scr or CHC KD shRNA were stimulated (40 Hz 5 s) as indicated by bar. Average time course $\Delta F/F_0$ ± SEM of the evoked syt1-pH response is displayed (n = 10 Scr, n = 9 CHC KD, ***=$p<0.001$, two-way ANOVA).
