# Supplementary material for: VAMP4 Is an Essential Cargo Molecule for Activity-Dependent Bulk Endocytosis
Source: Neuron. 2015 Dec 2;88(5):973–84. doi: 10.1016/j.neuron.2015.10.043 (PMC4678114; doi:10.1016/j.neuron.2015.10.043)
Supplement: Document S2. Article plus Supplemental Information [file mmc2.pdf]

Neuron

Article

VAMP4 Is an Essential Cargo Molecule for Activity-Dependent Bulk Endocytosis

Highlights

- VAMP4 is the first identified ADBE cargo
- VAMP4 is essential for ADBE
- Most synaptic vesicle cargoes are not selectively recovered by ADBE

Authors

Jessica C. Nicholson-Fish,
Alexandros C. Kokotos,
Thomas H. Gillingwater,
Karen J. Smillie, Michael A. Cousin

Correspondence

k.smillie@ed.ac.uk (K.J.S.),
m.cousin@ed.ac.uk (M.A.C.)

In Brief

Nicholson-Fish et al. show that VAMP4 is selectively retrieved by activity-dependent bulk endocytosis (ADBE) and is essential for ADBE to proceed. Thus, synaptic vesicles generated by ADBE will have a specific molecular composition that defines their physiological function.

CrossMark

Nicholson-Fish et al., 2015, Neuron *88*, 973–984
December 2, 2015 ©2015 The Authors
http://dx.doi.org/10.1016/j.neuron.2015.10.043

CellPress

CellPress

VAMP4 Is an Essential Cargo Molecule for Activity-Dependent Bulk Endocytosis

Jessica C. Nicholson-Fish,[1] Alexandros C. Kokotos,[1] Thomas H. Gillingwater,[1] Karen J. Smillie,[1,*] and Michael A. Cousin[1,*]
[1]Centre for Integrative Physiology, University of Edinburgh, Hugh Robson Building, George Square, Edinburgh EH8 9XD, Scotland
*Correspondence: k.smillie@ed.ac.uk (K.J.S.), m.cousin@ed.ac.uk (M.A.C.)
http://dx.doi.org/10.1016/j.neuron.2015.10.043
This is an open access article under the CC BY license (http://creativecommons.org/licenses/by/4.0/).

SUMMARY

The accurate formation of synaptic vesicles (SVs) and incorporation of their protein cargo during endocytosis is critical for the maintenance of neurotransmission. During intense neuronal activity, a transient and acute accumulation of SV cargo occurs at the plasma membrane. Activity-dependent bulk endocytosis (ADBE) is the dominant SV endocytosis mode under these conditions; however, it is currently unknown how ADBE mediates cargo retrieval. We examined the retrieval of different SV cargo molecules during intense stimulation using a series of genetically encoded pH-sensitive reporters in neuronal cultures. The retrieval of only one reporter, VAMP4-pHluorin, was perturbed by inhibiting ADBE. This selective recovery was confirmed by the enrichment of endogenous VAMP4 in purified bulk endosomes formed by ADBE. VAMP4 was also essential for ADBE, with a cytoplasmic di-leucine motif being critical for this role. Therefore, VAMP4 is the first identified ADBE cargo and is essential for this endocytosis mode to proceed.

INTRODUCTION

The efficient formation of synaptic vesicles (SVs) from the plasma membrane after neurotransmitter release is critical to maintain the fidelity of neurotransmission across a wide range of stimulation intensities. Distinct SV endocytosis modes are present within central nerve terminals that are triggered by discrete patterns of neuronal activity. These are ultrafast endocytosis (Watanabe et al., 2013, 2014), clathrin-mediated endocytosis (CME), which is prevalent during mild stimulation (Granseth et al., 2006), and activity-dependent bulk endocytosis (ADBE), which is only triggered during intense neuronal activity (Clayton et al., 2008; Clayton and Cousin, 2009a). The route for SV formation differs between these endocytosis modes, with CME generating single SVs, whereas both ultrafast and ADBE generate endosomes directly from the plasma membrane from which SVs can then bud (Kokotos and Cousin, 2015).

A key aspect in the formation of functional SVs is the incorporation of the correct protein cargo with the appropriate stoichiometry. CME utilizes both the adaptor protein complex AP-2 and a series of monomeric adaptor proteins to ensure a high fidelity of SV cargo clustering and incorporation (Diril et al., 2006; Kelly and Owen, 2011; Koo et al., 2011; Rao et al., 2012). In contrast, very little is known regarding the mechanism of SV cargo retrieval from the plasma membrane in either ultrafast endocytosis or ADBE. Recent studies have hinted that cargo sorting occurs primarily at the endosome for both modes (Cheung and Cousin, 2012; Kononenko et al., 2014; Watanabe et al., 2014), suggesting that endosomes are formed in a relatively non-specific manner and may resemble the plasma membrane in composition (Kononenko et al., 2014). During ADBE the generation of “bulk” endosomes is rapid and synchronous with neuronal activity (Clayton et al., 2008). This event is widely thought to be clathrin independent, since inactivation or knockdown of clathrin does not impact on either the formation of bulk endosomes (Heerssen et al., 2008; Kasprowicz et al., 2008) or the recovery of SV cargo during high-frequency stimulation (Kononenko et al., 2014).

We investigated whether directed sorting of SV cargo occurs at the plasma membrane during ADBE, since this will ultimately impact on the molecular composition of SVs generated via this endocytosis mode. We examined the retrieval of a series of exogenously expressed SV cargo molecules, and we found that only one was preferentially trafficked via ADBE—VAMP4. Endogenous VAMP4 was selectively enriched on bulk endosomes and was also essential for ADBE to proceed, indicating that it is an essential ADBE cargo molecule.

RESULTS

VAMP4 Is Selectively Retrieved from the Plasma Membrane by ADBE

ADBE is triggered by intense neuronal stimulation and is the dominant mode of SV retrieval during such stimuli (Clayton et al., 2008). However, little is known regarding SV cargo selection mechanisms by this endocytosis mode. To investigate this, we examined the trafficking of a series of genetically encoded reporters of SV cargo that have a pH-sensitive GFP moiety (pHluorin) fused to a luminal domain. Such reporters are widely used to monitor both SV fusion and CME, since pHluorin fluorescence is quenched in acidic environments such as the SV interior

CrossMark

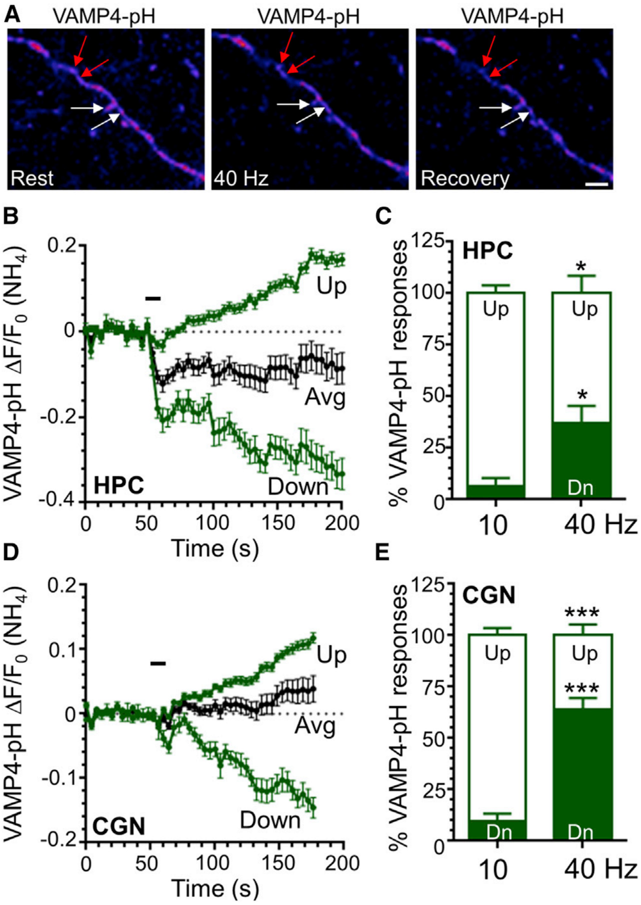

Figure 1. VAMP4-pHluorin Displays a Slow Fluorescent Downstroke Only after Intense Stimulation

(A) Representative images of VAMP4-pHluorin (VAMP4-pH) fluorescence in hippocampal (HPC) neurons in response to a train of 400 action potentials delivered at 40 Hz. Images are false colored with the panels indicating fluorescence at rest (left), during stimulation (40 Hz, middle), or 2 min after stimulation (Recovery, right). White arrows indicate nerve terminals that increase after the stimulation is complete, whereas red arrows show nerve terminals that continue to decrease. Scale bar, 10 μm.

(B and D) Hippocampal or cerebellar (CGN) neurons transfected with VAMP4-pH were stimulated at 40 Hz, 10 s (indicated by bar). The time course of the average (Avg) VAMP4-pH response in nerve terminals is displayed as $\Delta F/F_0$ ± SEM (normalized to the total pHluorin pool [NH_4]). This average trace can be dissected into two discrete populations that display either slow increases (Up) or decreases (Down) after stimulation in either (B) HPCs or (D) CGNs.

(C and E) HPCs (C) or CGNs (E) transfected with VAMP4-pH were stimulated with a train of either low-frequency (10 Hz, 30 s) or high-frequency (40 Hz, 10 s) stimulation. The percentage of Up (open bars) and down (Dn, solid bars) responses in individual nerve terminals in displayed, ± SEM (HPC: n = 4 [10 Hz, 40 Hz]; CGN: n = 7 [40 Hz], n = 5 [10 Hz]; ***$p < 0.001$; *$p < 0.05$; two-way ANOVA).

(Kavalali and Jorgensen, 2014). SV exocytosis is therefore reported as an increase in fluorescence, whereas SV endocytosis is monitored as a decrease, since CME is rate limiting when compared to the rate of SV acidification (Sankaranarayanan and Ryan, 2000; Atluri and Ryan, 2006) (but see Egashira et al., 2015). This interpretation becomes more complicated when monitoring ADBE, however, since the initial step in this pathway is the formation of a bulk endosome, which is present in nerve terminals for at least 30 min following its generation (Cheung et al., 2010). From first principles the larger interior of the bulk endosome when compared to a SV (approximately 50-fold for a 150 nm endosome) should mean it takes longer to acidify. This should retard the rate of the post-stimulation fluorescence decay, rendering interpretation of the pHluorin signal a much more complex process.

We first examined the trafficking of VAMP4-pHluorin during intense neuronal activity, since this reporter displays a unique activity-dependent fluorescent profile in comparison to other pHluorin-tagged SV cargo. VAMP4-pHluorin exhibits a fluorescence decrease when challenged with action potentials followed by a slow post-stimulation increase (Raingo et al., 2012), suggesting its activity-dependent retrieval from the plasma membrane occurs only during intense stimulation. The post-stimulation, activity-dependent increase is thought to be asynchronous release (Raingo et al., 2012), which is proposed to be mediated by SVs generated by ADBE (Evstratova et al., 2014); thus, VAMP4-pHluorin may be a potential ADBE cargo. When cultured hippocampal neurons expressing VAMP4-pHluorin were stimulated with a train of high-frequency action potentials (40 Hz, 10 s) to evoke ADBE, they displayed an average response consisting of an immediate downstroke and slow recovery to baseline (Figures 1A and 1B) (Raingo et al., 2012). However, when individual nerve terminal responses were assessed, the average response could be dissected into two discrete post-stimulation fluorescent profiles (Figure 1B). Approximately 40% of nerve terminals displayed a slow decrease in fluorescence after stimulation, whereas 60% displayed an increase (Figure 1C). We next assessed the evoked VAMP4-pHluorin profile in cultures of cerebellar neurons since a larger proportion of their nerve terminals display ADBE (Clayton and Cousin, 2009b), most likely due to chronic stimulation in culture (Burgoyne and Cambray-Deakin, 1988). In these neurons 65% of nerve terminals displayed a slow fluorescent decrease and only 35% displayed an increase, correlating with the higher prevalence of ADBE in this culture system (Figures 1D and 1E).

We hypothesized that this slow fluorescent downstroke may represent the acidification of bulk endosomes after their rapid activity-dependent generation by ADBE. We tested this by performing a series of corroborating experiments. First we determined the number of nerve terminals displaying slow VAMP4-pHluorin downstrokes in response to 10 Hz stimulation, a protocol that primarily triggers CME and not ADBE (Clayton et al., 2008). Very few nerve terminals displayed a VAMP4-pHluorin downstroke in either hippocampal or cerebellar neurons under these stimulation conditions (Figures 1C and 1E). Thus, when ADBE is not triggered by intense stimulation, the number of slow VAMP4-pHluorin downstrokes is negligible.

To determine whether slow VAMP4-pHluorin downstrokes reflect bulk endosome acidification after ADBE, we examined how the fluorescent response is altered when this endocytosis mode was inhibited. We employed a stimulation 1 (S1) and stimulation 2 (S2) protocol in cerebellar cultures, where two identical high-frequency trains (40 Hz, 10 s) were applied 10 min apart (Figures 2A and 2B). A very similar proportion of nerve terminals

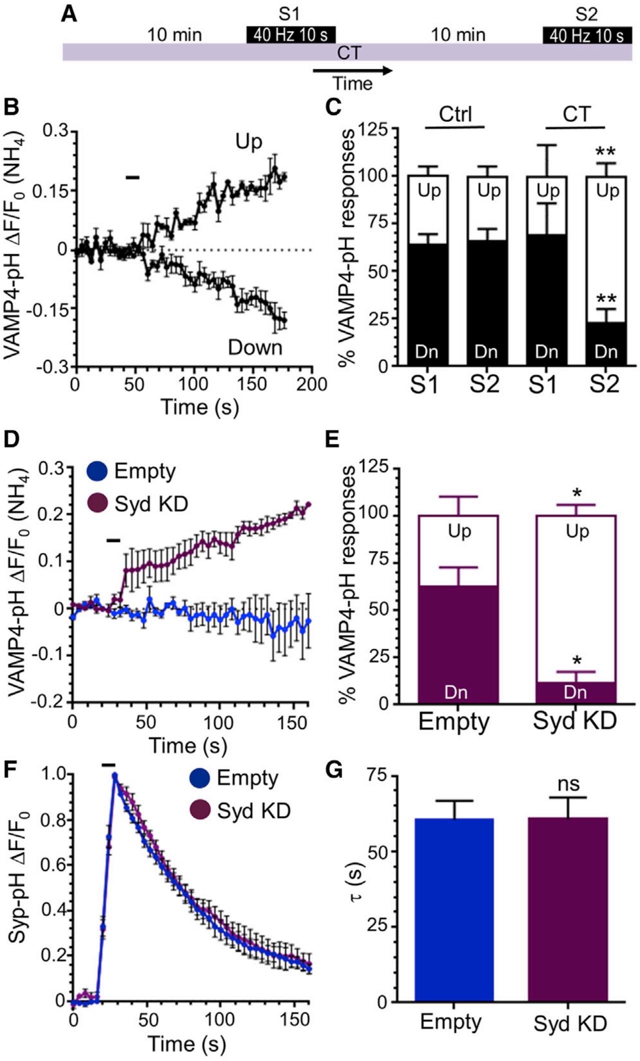

Figure 2. Slow VAMP4-pHluorin Downstrokes Are Arrested during Inhibition of ADBE

(A) Cerebellar neurons transfected with VAMP4-pHluorin (VAMP4-pH) were stimulated with two sequential action potential trains (S1 and S2) 10 min apart (both 40 Hz, 10 s). Cultures were incubated with 2 μM CT99021 (CT) 10 min prior to S1 and then continuously onward where indicated.

(B) Representative time course of the control S1 VAMP4-pH response dissected into fluorescent upstrokes (Up) or downstrokes (Down) is displayed as $\Delta F/F_0$ ± SEM (normalized to the total pHluorin pool [NH_4]). The stimulation is indicated by the bar.

(C) The percentage of Up (open bars) and Down (Dn, solid bars) responses in individual nerve terminals in either the absence (Ctrl) or presence of CT99021 (CT) are displayed ± SEM (n = 7 Ctrl; n = 5 CT; **$p < 0.01$; two-way ANOVA).

(D–G) Cerebellar neurons were transfected with either empty shRNA or shRNA against syndapin I and either (D and E) VAMP4-pHluorin (VAMP4-pH) or (F and G) synaptophysin-pHluorin (syp-pH). Cultures were stimulated with an action potential train (40 Hz, 10 s).

(D and F) Average time course in neurons expressing either empty vector (Empty, blue) or shRNA against syndapin I (Syd KD, purple) is displayed as $\Delta F/F_0$ ± SEM (normalized to the total pHluorin pool [NH_4] for VAMP4-pH and peak fluorescence for syp-pH). The bar indicates the period of stimulation.

(E) The percentage of Up (open bars) and Down (Dn, solid bars) VAMP4-pH responses in individual nerve terminals are displayed, ± SEM (n = 5 Empty; n = 4 Syd KD; *$p < 0.05$; two-way ANOVA).

(F) Quantification of the average time constant (τ) ± SEM of the evoked syp-pH response (n = 8 Empty; n = 5 Syd KD; ns, non-significant; Student's t test).

displaying evoked slow downstrokes was observed at both S1 and S2, indicating the reproducibility of the response (Figure 2C). We assessed the effect of arresting ADBE by inhibiting glycogen synthase kinase 3 (GSK3). Antagonism of this protein kinase has no effect on ADBE during an initial high-frequency stimulus; however, ADBE is inhibited during subsequent identical action potential challenges (Evans and Cousin, 2007; Clayton et al., 2010). This was confirmed by monitoring uptake of large fluorescent dextran molecules (tetramethylrhodamine-dextran [TMR-dextran]) that selectively report ADBE (Figure S1A). When S1 and S2 experiments were performed in the presence of the GSK3 antagonist CT99021 (2 μM), there was no difference in the proportion of nerve terminals displaying slow VAMP4-pHluorin downstrokes during the first stimulus train (Figure 2C). However, when S2 responses were monitored (at which time ADBE is inhibited), there was a significant reduction in the number of nerve terminals displaying a downward fluorescent response (Figure 2C). Thus, inhibition of ADBE greatly diminishes the proportion of nerve terminals displaying activity-dependent slow VAMP4-pHluorin downstrokes, suggesting this downstroke reflects bulk endosome acidification.

To confirm that slow VAMP4-pH fluorescent downstrokes were ADBE dependent, we silenced expression of the essential ADBE molecule syndapin I using a characterized short hairpin RNA (shRNA) vector (Clayton et al., 2009). Inhibition of ADBE was confirmed by monitoring TMR-dextran uptake during high-frequency stimulation (Figure S1C). Syndapin I knockdown dramatically altered the average VAMP4-pHluorin response, with almost all nerve terminals displaying an immediate evoked increase in signal (Figure 2D). Indeed, syndapin I knockdown reduced the number of nerve terminals displaying slow VAMP4-pHluorin downstrokes to almost zero (Figure 2E). This provides compelling evidence that ADBE is responsible for these evoked downstrokes during intense neuronal activity.

If VAMP4-pHluorin is specifically retrieved via ADBE, its fluorescent signal should be inaccessible to impermeant weak acid, since it will be trapped inside a slowly acidifying bulk endosome directly after intense stimulation. To test this, an impermeant acid solution was applied immediately after high-frequency (40 Hz) stimulation in either cerebellar or hippocampal neurons to quench the fluorescent signal from the surface reporter. Approximately 40% of the VAMP4-pHluorin response was resistant to quenching when compared to a pre-stimulus baseline in either neuronal subtype (Figures 3A and 3B). Importantly, complete quenching of the VAMP4-pHluorin response was observed at 10 Hz stimulation, where ADBE is not triggered (Figure 3B). This indicates that VAMP4-pHluorin is located inside a slowly acidifying compartment such as a bulk endosome after intense stimulation and that VAMP4 is a bone fide cargo for ADBE.

Most SV Cargoes Are Not Selectively Recovered by ADBE

To determine whether other SV cargoes are also recovered by ADBE, we performed a surface quenching experiment in cerebellar neurons using synaptophysin-pHluorin (syp-pHluorin) as

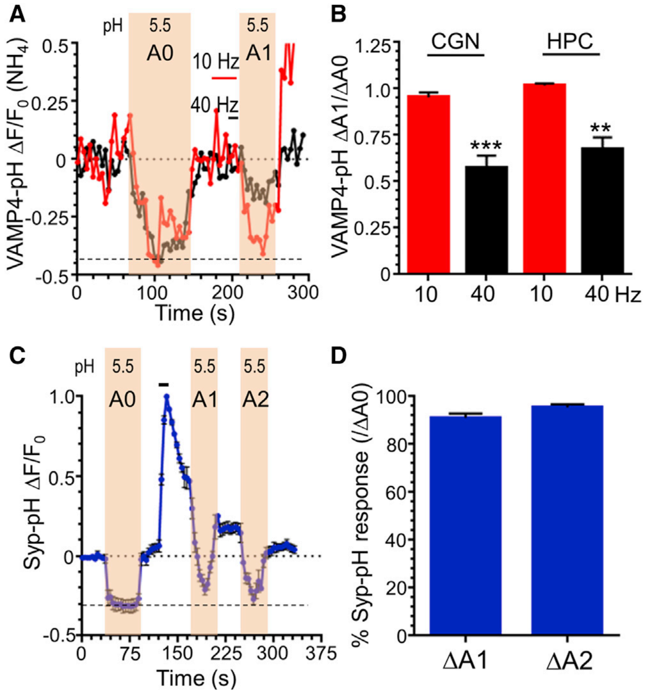

Figure 3. VAMP4-pHluorin Fluorescence Is Inaccessible to Acid after Intense Stimulation

(A) Cerebellar (CGN) or hippocampal (HPC) neurons transfected with VAMP4-pHluorin (VAMP4-pH) were stimulated with either low-frequency (10 Hz, 30 s; red) or high-frequency (40 Hz, 10 s; black) stimulation indicated by bar. The representative VAMP4-pH response in CGNs during challenge with acidic buffer (illustrated by shaded regions) either before (A0) or directly after (A1) stimulation is displayed. The dotted line illustrates the baseline signal during exposure to acid.

(B) Quantification of the accessibility of acid solution after stimulation (ΔA1/ΔA0) in both CGN and HPC neurons (CGN: n = 5 [10 Hz], n = 4 [40 Hz]; HPC: n = 3 [10 Hz], n = 4 [40 Hz]; $^{**}p < 0.01$; $^{***}p < 0.001$; Student's t test).

(C) CGNs transfected with synaptophysin-pHluorin (syp-pH) were stimulated for 10 s at 40 Hz (indicated by bar). The average syp-pH response during challenge with acidic buffer (illustrated by shaded regions) either before (A0) or after stimulation (A1 and A2) is displayed as $\Delta F/F_0$ ± SEM. The dotted line illustrates the baseline signal during exposure to acid.

(D) Quantification of the accessibility of acid solution at either pulse 1 (ΔA1/ΔA0) or 2 (ΔA2/ΔA0) as a percentage of total quenchable syp-pH fluorescence (n = 12; ns; one-way ANOVA).

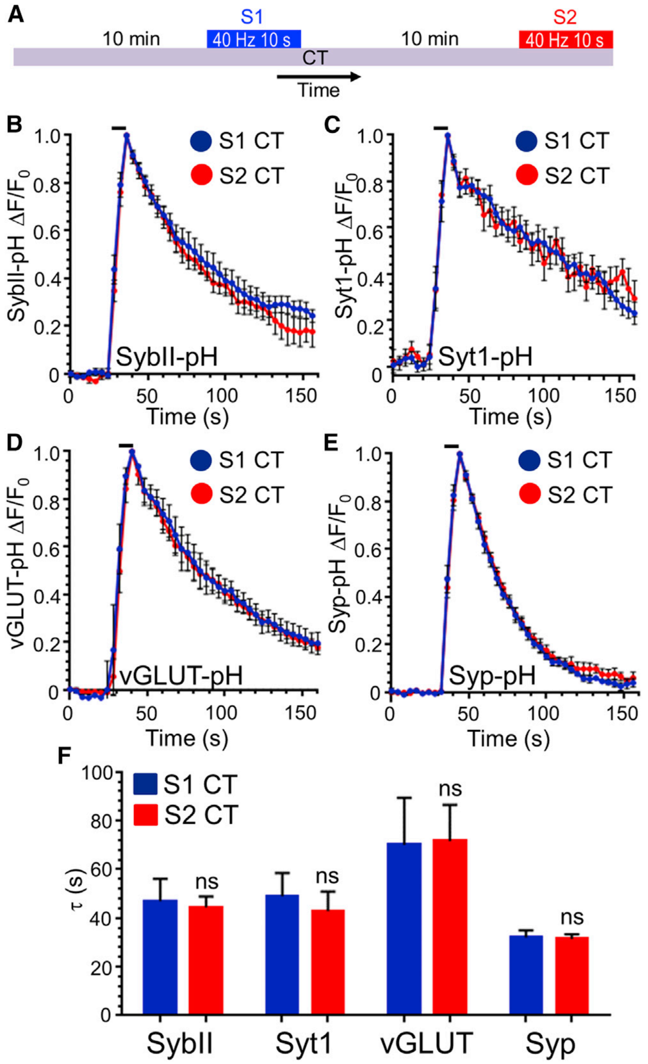

Figure 4. Inhibition of ADBE Does Not Affect the Retrieval of Multiple SV Cargo Molecules

(A) Cerebellar neurons transfected with the pHluorin reporters synaptobrevin II-pHluorin (sybII-pH), synaptotagmin-1-pHluorin (syt1-pH), vGLUT-pHluorin (vGLUT-pH), or synaptophysin-pHluorin (syp-pH) were stimulated with two sequential action potential trains (S1 and S2) 10 min apart (both 40 Hz, 10 s). Cultures were incubated with 2 μM CT99021 (CT) 10 min prior to S1 and then continuously onward.

(B–E) Average time course of the fluorescent response of either sybII-pH (B), syt1-pH (C), vGLUT-pH (D), or syp-pH (E) presented as $\Delta F/F_0$ ± SEM at both S1 (blue) and S2 (red). In all cases, the bar indicates the period of stimulation.

(F) Quantification of the average time constant (τ) ± SEM of the evoked sybII-pH (Syb), syt1-pH (Syt1), vGLUT-pH (vGLUT), and syp-pH (Syp) response for both S1 (blue) and S2 (red) traces (n = 4 sybII-pH; n = 10 syt1-pH; n = 5 vGLUT-pH; n = 6 syp-pH; ns; one-way ANOVA).

a typical SV cargo. In contrast to VAMP4-pHluorin, application of acid pulses immediately after high-frequency stimulation decreased the syp-pHluorin response to near baseline levels (Figures 3C and 3D). Thus, virtually all syp-pHluorin fluorescence is present inside rapidly acidifying compartments (such as SVs) immediately after high-frequency stimulation. This suggests that syp-pHluorin is not recovered by ADBE during intense neuronal activity.

To determine whether a series of other SV cargo molecules could be recovered by ADBE, we performed S1 and S2 experiments in cerebellar neurons with pHluorin-tagged versions of either syp-pHluorin, synaptobrevin II (sybII-pHluorin), synaptotagmin-1 (syt1-pHluorin), or the vesicular glutamate transporter (vGLUT1-pHluorin) (Figure 4A). In all cases the evoked fluorescent response was unaltered between the S1 and S2 challenge, in terms of the extent of the fluorescent response or the kinetics of fluorescence recovery (Figure S2). When these experiments were repeated in the presence of CT99021, there was no change in either the extent of the pHluorin signal (Figure S3B) or the kinetics of fluorescence recovery (Figures 4B–4F) between S1

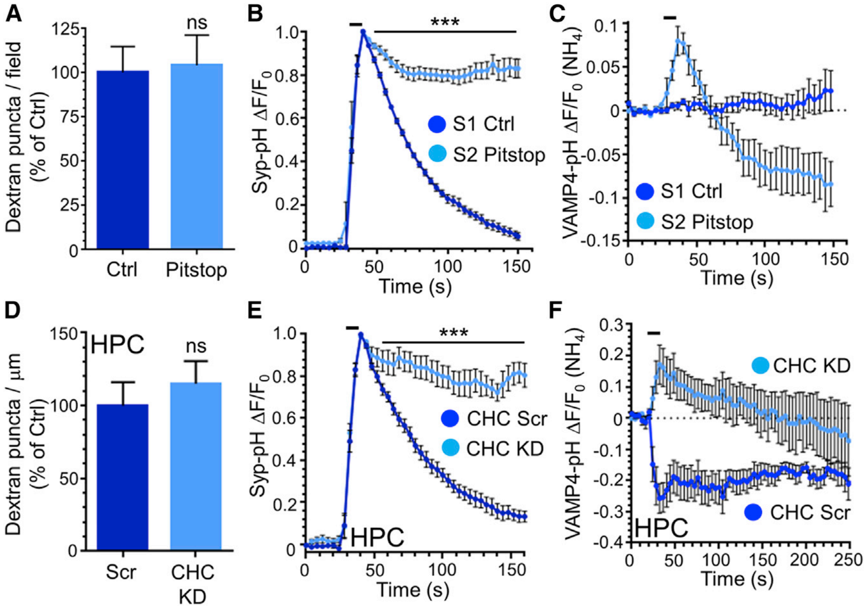

Figure 5. CME Inhibition Arrests SV Cargo Retrieval Except VAMP4-pHluorin during Intense Stimulation

(A) Cerebellar neurons were stimulated (40 Hz, 10 s) in the presence of 50 μM TMR-dextran with or without (Ctrl) 15 μM pitstop-2. Quantification of TMR-dextran puncta per field ± SEM is normalized to Ctrl (n = 7 for both; ns; Student's t test).

(B and C) Cerebellar neurons transfected with either synaptophysin-pHluorin (syp-pH) or VAMP4-pHluorin (VAMP4-pH) were stimulated (40 Hz, 10 s) in the presence or absence of 15 μM pitstop-2. The average time course $\Delta F/F_0$ ± SEM is displayed for syp-pH (B) and VAMP4-pH (C) for Ctrl (S1, dark blue) and pitstop-2 (S2, light blue) neurons (syp-pH: n = 6; VAMP4-pH: n = 3; ***$p < 0.001$; two-way ANOVA).

(D) Hippocampal (HPC) neurons transfected with either scrambled (Scr) or shRNA against clathrin heavy chain (CHC KD) were stimulated (40 Hz, 10 s) in the presence of 50 μM TMR-dextran. Quantification of dextran puncta per μm ± SEM is normalized to Scr control (n = 20 Scr; n = 17 CHC; ns; Student's t test).

(E and F) HPCs transfected with either syp-pH or VAMP4-pH and either Scr or CHC KD shRNA were stimulated (40 Hz, 10 s) as indicated by bar. The average time course $\Delta F/F_0$ ± SEM is displayed for syp-pH (E) and VAMP4-pH (F) for Scr control (dark blue) and CHC KD (light blue) neurons (syp-pH: n = 9 Scr, n = 6 CHC KD; VAMP4-pH: n = 5 Scr, n = 4 CHC KD; ***$p < 0.001$; two-way ANOVA).

and S2 for any reporter. This suggests that inhibition of ADBE does not impact on the recovery of most SV cargoes during intense stimulation, a premise supported by the fact that a second maneuver that arrests ADBE (inhibition of cyclin-dependent kinase 5 with the antagonist roscovitine; Figure S1B) also had no effect on activity-dependent syp-pHluorin retrieval (Figures S3C and S3D). This was confirmed by the absence of effect on the evoked syp-pHluorin response in cerebellar neurons where expression of the ADBE molecule syndapin I had been silenced (Figures 2F and 2G; Figure S1D). Thus, inhibition of ADBE does not affect the trafficking of exogenously expressed SV cargo.

To ensure that the lack of retrieval of typical SV cargo by ADBE was not specific to cerebellar neurons, we repeated this experiment in primary cultures of hippocampal neurons expressing syp-pHluorin. These neurons responded to both stimulus trains in a very similar manner to cerebellar neurons (Figure S4B). When incubated with CT99021 to inhibit ADBE during the second stimulus train, there was no difference in either the extent of syp-pHluorin response or the kinetics of its fluorescence recovery (Figures S4C and S4D) compared to untreated neurons. Thus, inhibiting ADBE does not impact on the trafficking of a series of exogenously expressed SV cargo molecules during intense neuronal activity in multiple culture systems.

Most SV Cargoes Are Retrieved by CME during High-Intensity Stimulation

We have shown that VAMP4-pHluorin is selectively recovered by ADBE during intense stimulation. However, the endocytosis mode responsible for recovery of other SV cargo remains unclear. CME is maximally active during intense stimulation (Clayton et al., 2008) and therefore may be responsible for the recovery of these cargo molecules. To test this, we examined the trafficking of syp-pHluorin after inhibition of CME in cerebellar neurons. CME was arrested by exposure to the clathrin inhibitor pitstop-2 (15 μM) (von Kleist et al., 2011). Pitstop-2 had no effect on ADBE, confirmed by its absence of effect on TMR-dextran uptake evoked by frequency stimulation (Figure 5A). In contrast, pitstop-2 abolished the fluorescence recovery of syp-pHluorin after an identical stimulus train (Figure 5B). Thus, syp-pHluorin appears to be predominantly trafficked via CME even during intense neuronal activity. Prolonged exposure to pitstop-2 deacidifies SVs (Hua et al., 2013), potentially explaining the arrest of the syp-pHluorin fluorescence recovery. However, this effect only became apparent beyond the timescale of our experiments; therefore, SV deacidification is not responsible for this block of syp-pHluorin retrieval (Figure S5).

We next determined whether arresting CME interfered with the evoked VAMP4-pHluorin response during high-frequency stimulation. We predicted that the fluorescent downstroke would not be affected by this maneuver, since it would be independent of CME. This was the case, with the VAMP4-pHluorin response displaying an initial upstroke followed by a slow post-stimulation fluorescence decrease to below baseline levels when CME was arrested using pitstop-2 (Figure 5C). This response was observed in all nerve terminals investigated. Thus, the slow VAMP4-pHluorin downstroke is unaffected by the arrest of CME, confirming its dependence on ADBE.

To confirm these observations, we knocked down expression of clathrin heavy chain (CHC) using shRNA oligonucleotides (Royle et al., 2005). This shRNA approach reduced endogenous CHC levels in cerebellar neurons, but surprisingly it also inhibited TMR-dextran uptake (Figures S6A and S6C). This inhibition was most likely due to chronic arrest of clathrin-dependent SV

budding from bulk endosomes, since cerebellar neurons are cultured in permanently depolarizing conditions (Burgoyne and Cambray-Deakin, 1988). In agreement, CHC knockdown had no effect on TMR-dextran uptake in cultured hippocampal neurons (Figure 5D). We therefore examined the effect of CHC knockdown on syp-pHluorin retrieval in hippocampal neurons, since its effect in cerebellar neurons cannot be interpreted due to off-target effects on ADBE (Figure S6C). Neurons expressing scrambled shRNA exhibited a characteristic syp-pHluorin response, whereas those expressing CHC shRNA exhibited a greatly retarded fluorescence recovery after stimulation (Figure 5E). Thus, inhibition of CME by either CHC knockdown or a clathrin inhibitor significantly impacts on syp-pHluorin retrieval during high-frequency stimulation, indicating that CME is the dominant SV retrieval mode for this SV cargo even during intense neuronal activity. We also determined the effect of CHC knockdown on the VAMP4-pHluorin response in hippocampal neurons. Neurons expressing CHC shRNA displayed a similar VAMP4-pHluorin profile to pitstop-2-treated neurons, with an initial increase followed by a slow post-stimulation downstroke (Figure 5F). Thus, the slow VAMP4-pHluorin downstroke, which is only triggered during high intensity, was unaffected by two independent maneuvers that arrest CME, confirming its dependence on ADBE.

Endogenous VAMP4 Is Retrieved by ADBE

We have shown that VAMP4-pHluorin is a genetically encoded reporter of ADBE. We next determined whether endogenous VAMP4 was also selectively accumulated by ADBE. To achieve this we performed biochemical fractionation experiments to enrich bulk endosomes from neuronal cultures. Cerebellar neurons are excellent for such studies, since they can be cultured to greater than 95% homogeneity (Burgoyne and Cambray-Deakin, 1988). We confirmed the enrichment of a bulk endosome fraction by tracking newly formed endosomes and SVs using the fluorescent dye FM1-43. After loading FM1-43 in the absence or presence of a strong stimulus, the neurons were lysed by mechanical disruption. The post-nuclear supernatant was then fractionated using discontinuous Nycodenz gradients after a short spin to remove cellular debris (Figure 6A) (Barysch et al., 2010). Analysis of FM1-43 fluorescence in the stimulated fractions showed two distinct peaks, corresponding to endosomes (fraction 2) and SVs (fraction 5) (Figure 6B) (Barysch et al., 2010). To confirm that these fractions were enriched for bulk endosomes and SVs, respectively, we performed an identical experiment using the fluid phase marker horse radish peroxidase (HRP). Morphological analysis confirmed the enrichment of HRP-labeled endosomes in fraction 2 and HRP-labeled SVs in fraction 5 when comparing stimulated to unstimulated cultures (Figures 6C–6G).

Next we probed for the presence of endogenous SV cargo by western blotting. This was achieved by comparing the presence of these cargoes in endosome and SV fractions from the same preparation of stimulated cultures. The endogenous SV cargoes syt1, syp, sybII, and vGLUT were all present in both the bulk endosome and SV fraction (Figures 6H and 6I). In contrast, VAMP4 was almost exclusively present in the endosome fraction (Figures 6H and 6I). Thus, bulk endosomes do contain endogenous SV cargo; however, only VAMP4 is enriched within this compartment.

To confirm the localization of VAMP4 to bulk endosomes in vivo, we performed ultrastructural analyses using silver-enhanced immunogold staining in either resting or stimulated (40 Hz, 10 s) cerebellar cultures. We observed a high degree of VAMP4 localization to bulk endosomes in stimulated nerve terminals, confirming its recovery via ADBE (Figure 7A).

VAMP4 Is Required for ADBE

One key remaining question is whether VAMP4 simply represents an ADBE cargo or whether it is essential for ADBE to proceed. We determined this by using validated shRNA oligonucleotides against VAMP4 (Bal et al., 2013) (Figures S7A–S7C). Knockdown of VAMP4 had no effect on the syp-pHluorin response evoked by 40Hz stimulation, indicating no essential role in CME (Figure S7D). However, under identical stimulation conditions VAMP4 knockdown abolished TMR-dextran uptake, an inhibition fully rescued by expression of wild-type VAMP4-pHluorin (Figures 7B and 7C). Therefore, in addition to being the first identified ADBE cargo molecule, VAMP4 is essential for this key endocytosis mode.

The essential requirement for VAMP4 in ADBE suggests it must share key interactions with other endocytosis molecules to direct this process. One potential association is with adaptor proteins, since a di-leucine motif on the cytoplasmic N terminus of VAMP4 coordinates such interactions (Peden et al., 2001). To test this we performed a rescue experiment in VAMP4 knockdown neurons using a VAMP4 mutant (L25A) that disrupts this interaction (Peden et al., 2001; Raingo et al., 2012). We observed no rescue of TMR-dextran uptake with this mutant (Figures 7B and 7C), suggesting interactions between VAMP4 and adaptor proteins are essential for progression of ADBE.

We next examined whether disrupted adaptor protein interactions affected the trafficking of VAMP4-pHluorin. This mutation abolished the fast activity-dependent VAMP4-pHluorin downstroke in both cerebellar and hippocampal neurons in agreement with previous work (Raingo et al., 2012) (Figures 8A and 8B). Furthermore, the L25A mutant displayed a similar average trafficking profile to wild-type VAMP4-pHluorin in neurons where ADBE had been inhibited (compare Figures 8A and 8B with Figure 2C). This effect was not due to a dominant-negative effect on ADBE by L25A overexpression, since overexpression of this mutant or wild-type VAMP4-pHluorin has no effect on evoked TMR-dextran uptake (Figure S7E). Thus, adaptor interactions of VAMP4 are essential for both its trafficking via ADBE and for ADBE itself.

The essential requirement for VAMP4 in ADBE provided us an opportunity to examine how ablation of this endocytosis mode alters presynaptic function during sustained and intense neuronal activity. To achieve this, we examined the trafficking of syp-pHluorin in response to four consecutive trains of high-frequency action potentials (40 Hz, 10 s) in hippocampal neurons transfected with either VAMP4 shRNA or a scrambled control. The syp-pHluorin response in control neurons was highly reproducible, displaying a reduction in the peak fluorescent response with each consecutive action potential train, presumably due to short-term depletion of SVs (Figures 8C

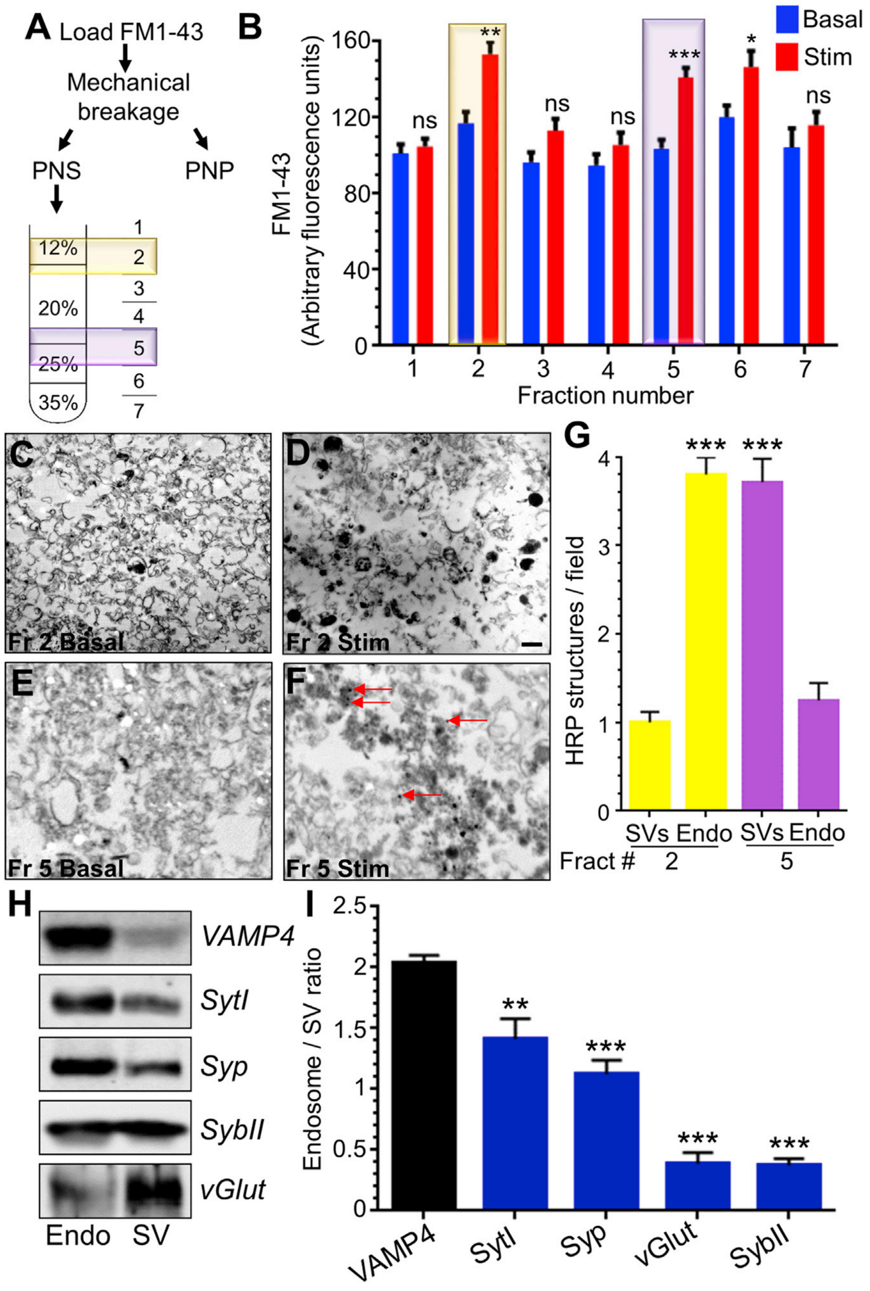

Figure 6. Endogenous VAMP4 Is Selectively Accumulated via ADBE

(A) Cerebellar neurons were stimulated with 50 mM KCl for 2 min in presence of 10 μM FM1-43. Cells were washed, mechanically broken, and centrifuged to separate intracellular compartments from broken cells and nuclei (PNP, post nuclear pellet). The post nuclear supernatant (PNS) was centrifuged though discontinuous Nycodenz gradients as illustrated. The fractions collected are illustrated, with the endosome (2) and SV (5) fractions highlighted.

(B) FM1-43 fluorescence from either basal (blue bars) or stimulated (red bars) fractions is presented as arbitrary fluorescence ± SEM (n = 6; Student's t test; ***$p < 0.001$; **$p < 0.01$; *$p < 0.05$). Fraction 2 (endosomes) and fraction 5 (SVs) are highlighted.

(C–G) An identical procedure was performed to load neurons with 10 mg/ml HRP, with fractions 2 (endosomes) and 5 (SVs) processed for electron microscopy. HRP-labeled structures greater than 80 nm were abundant in stimulated (Stim) (D), but not basal (C), samples from fraction 2 (Fr 2), whereas HRP-labeled SVs (30–60 nm) were abundant in stimulated (F), but not basal (E), samples from fraction 5 (Fr 5). Scale bar, 250 nm for all images. (G) Quantification of the evoked number of HRP-labeled structures per field. Yellow bars represent fraction 2, and purple bars represent fraction 5 (n = 15; Student's t test; SVs versus endosomes [Endo]; ***$p < 0.001$).

(H) Stimulated endosome and SV fractions were separated by SDS-PAGE and transferred to nitrocellulose membranes. Representative immunoblots for VAMP4, synaptotagmin-1 (Syt1), synaptophysin (Syp), synaptobrevin II (SybII), and vGLUT for endosome (Endo) and SVs fractions are shown.

(I) Quantification of the ratio of SV cargo between endosomes and SVs normalized for total protein content ± SEM (n = 3; one-way ANOVA to VAMP4; ***$p < 0.001$; **$p < 0.01$).

and 8D). VAMP4 knockdown neurons displayed a greater reduction in peak height during the latter stimulus trains when compared to the scrambled shRNA controls (Figures 8C and 8D). Importantly, the time constant of syp-pHluorin retrieval was not significantly different between VAMP4 knockdown and control neurons (Figure 8E), indicating that the reduction in peak height was not due to modulation of CME. Therefore, the reduction in presynaptic performance in VAMP4 knockdown neurons is most likely due to a reduction in SVs generated via ADBE, highlighting the importance of this endocytosis mode in maintaining neurotransmission during periods of sustained neuronal activity.

DISCUSSION

During brief bursts of intense neuronal activity, SV cargo and membrane transiently accumulate at the plasma membrane of central nerve terminals. ADBE is the dominant SV endocytosis mode under these conditions; however, it was unclear whether ADBE actively sorted cargo at the plasma membrane, since both clathrin and adaptor proteins are essential for SV generation from bulk endosomes (Heerssen et al., 2008; Kasprowicz et al., 2008; Cheung and Cousin, 2012; Kononenko et al., 2014). We have identified one SV cargo, VAMP4, which is specifically sorted into endosomes during ADBE and is also essential for ADBE to occur.

The generation of bulk endosomes via ADBE is a clathrin-independent process (Heerssen et al., 2008; Kasprowicz et al., 2008). Recent studies using syt1-pHluorin appeared to support this idea, since neither AP-2 nor clathrin knockdown had no effect

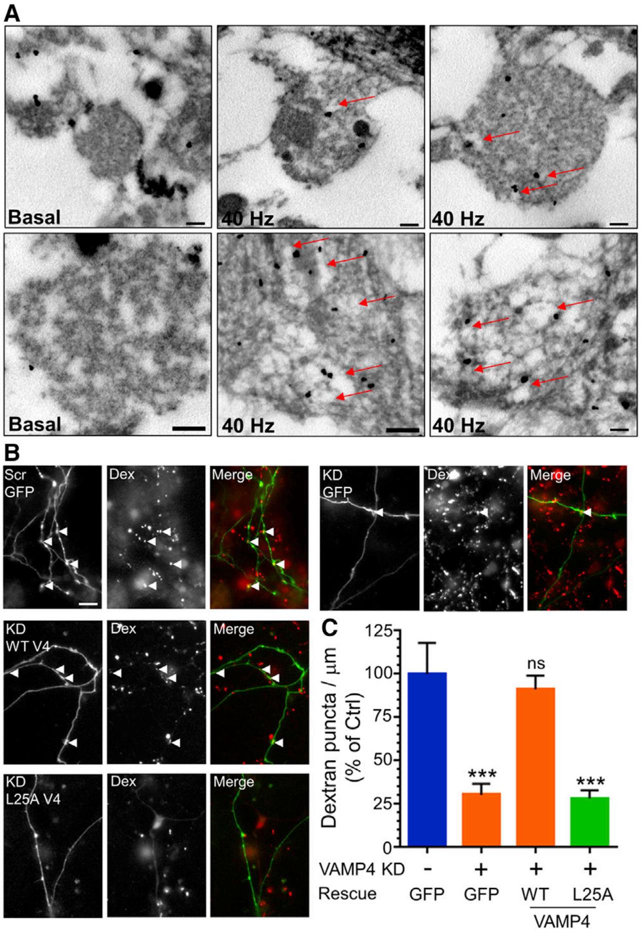

Figure 7. VAMP4 Is Essential for ADBE

(A) Cerebellar neurons were either left to rest (Basal) or challenged with an action potential stimulus (40 Hz, 10 s) and then processed for immunoelectron microscopy. Representative images show nerve terminals labeled with VAMP4 antibodies after immunogold detection and silver enhancement. Almost all silver particles were localized to endosomal structures after 40 Hz stimulation (indicated by arrows). Scale bars, 200 nm in all cases.

(B) Cerebellar neurons were transfected with either scrambled shRNA (Scr) or VAMP4 shRNA (KD) and co-expressed with either GFP, wild-type (WT V4), or L25A VAMP4-pHluorin (L25A V4). Neurons were stimulated with a train of action potentials (40 Hz, 10 s) in the presence of 50 µM TMR-dextran (Dex). Images display transfected neuron (green), TMR-dextran uptake (red), and a merged image for all experimental conditions. Arrowheads indicate nerve terminals that accumulated TMR-dextran. Scale bar, 15 µm.

(C) Quantification of dextran puncta per µm ± SEM normalized to Scr control (n = 23 Scr; n = 21 VAMP4 KD + GFP; n = 23 VAMP4 KD + WT VAMP4-pH; n = 26 VAMP4 KD + L25A VAMP4-pH; ***p < 0.001; one-way ANOVA).

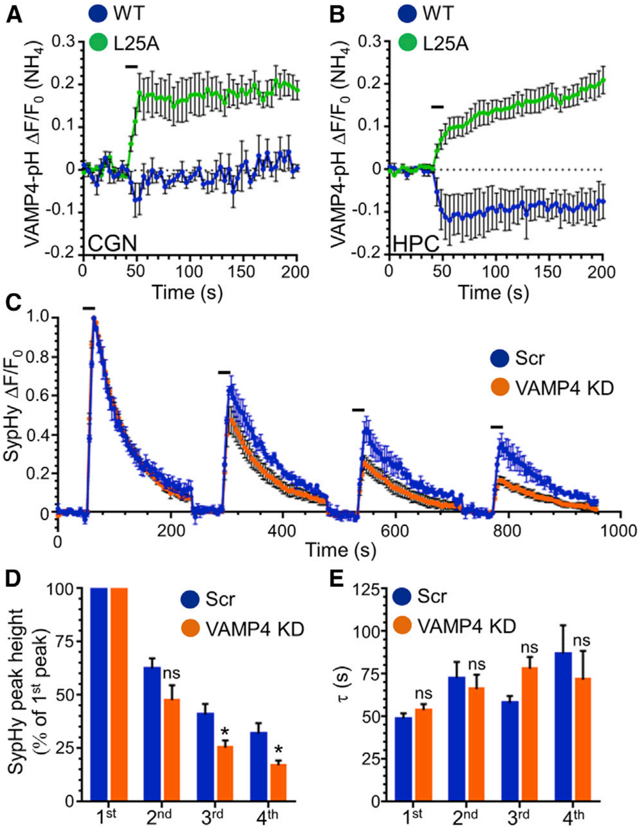

Figure 8. VAMP4 Is Required to Maintain Neurotransmission

(A and B) Cerebellar (CGN) (A) or hippocampal (HPC) (B) neurons were transfected with either wild-type (WT) VAMP4-pHluorin (VAMP4-pH) or an L25A mutant. Cultures were stimulated with an action potential train (40 Hz, 10 s) as indicated by the bar in the displayed average time course ($\Delta F/F_0$ ± SEM, normalized to the total pHluorin pool [(NH_4)]; CGN: both n = 5; HPC: n = 6 WT, n = 7 L25A).

(C) HPCs were transfected with syp-pH and either scrambled (Scr) or VAMP4 shRNA (KD). Neurons were challenged with four trains of action potential stimuli (40 Hz, 10 s) separated by 4 min as indicated by the bars. The average time course $\Delta F/F_0$ ± SEM is displayed for syp-pH for Scr control (blue) and VAMP4 KD (orange) neurons (n = 4 Scr; n = 5 VAMP4 KD).

(D and E) Quantification of either the syp-pH peak height normalized to the first peak (D) or the time constant (τ) (E) for each stimulus is displayed ± SEM (n = 4 Scr; n = 5 VAMP4 KD; *$p < 0.05$; Student's t test).

on the evoked fluorescent response during high-frequency stimulation (Kononenko et al., 2014). In contrast, we observed a robust inhibition of the syp-pHluorin response on inhibition of CME during intense activity using either the clathrin antagonist pitstop-2 or CHC knockdown. One potential explanation for this discrepancy is the efficiency of CHC knockdown by lentiviral shRNA (as used in Kononenko et al. [2014]), since this method displays a wide range of knockdown between individual cultured neurons (López-Murcia et al., 2014). Another confounding factor would be the temperature at which experiments were performed, since CME may not be dominant at 37°C (Watanabe et al., 2014). However, at physiological temperatures we still observed a robust arrest of the syp-pHluorin response with CHC knockdown (Figure S6E). Finally, under stimulation conditions identical to those in the previous study (Kononenko et al., 2014), we observed a robust inhibition of Syt1-pHluorin retrieval in cultured hippocampal neurons on CHC knockdown (Figure S8). We are currently unable to explain why we observe such disparate results using almost identical tools; however, we have shown identical effects on SV cargo retrieval in multiple systems using two independent maneuvers to arrest CME.

An important point to note from our biochemical enrichment of bulk endosomes is that endogenous SV cargoes were

accumulated by ADBE. This may be a result of ADBE accumulating excess SV cargo in a non-specific manner, due to CME reaching saturation capacity. An alternative explanation is that a population of SVs generated via CME may fuse with bulk endosomes to provide key molecules required for functional ADBE-derived SVs. Regardless, the presence of essential SV cargo on bulk endosomes will ensure that ADBE-derived SVs have the requisite complement of trafficking and fusion molecules that are essential for a functional SV.

We have identified the first genetically encoded reporter of ADBE, VAMP4-pHluorin. ADBE is visualized as a slow downstroke in VAMP4-pHluorin fluorescence after challenged with a train of high-frequency action potentials. This downstroke reflects acidification of bulk endosomes rather than VAMP4-pHluorin retrieval, since (1) ADBE only occurs during stimulation (Clayton et al., 2008) and (2) the downstroke continues for minutes after termination of stimulation. We also observed an immediate drop in VAMP4-pHluorin fluorescence in hippocampal neurons and to a lesser extent in cerebellar neurons, consistent with a parallel retrieval of this reporter via CME (Raingo et al., 2012). In agreement this fast activity-dependent fluorescent decrease was ablated by inhibition of this endocytosis mode. It was also ablated by mutating the interaction motif for adaptor proteins on VAMP4 (Raingo et al., 2012), suggesting that interactions mediated via this motif are also required for VAMP4 retrieval via CME. It should also be stated that CME is not required for retrieval of VAMP4 via ADBE, since the slow post-stimulation downstrokes were retained when CME was inhibited. Thus, an adaptor-dependent, clathrin-independent retrieval process retrieves VAMP4 as a first, essential step of ADBE.

The endosomal location of VAMP4 was confirmed by both in vivo ultrastructural analysis and the inability of impermeant acid to quench VAMP4-pHluorin fluorescence after high-intensity stimulation. This latter result meant that we were able to estimate the kinetics of bulk endosome acidification for the first time. We found VAMP4-pHluorin fluorescence decreased with a time constant of approximately 32 ± 2 s in the presence of pitstop-2 (to eliminate contaminating CME). This is an order of magnitude slower than SV acidification (Atluri and Ryan, 2006; Egashira et al., 2015), consistent with the larger internal volume of the bulk endosome compared to a SV (approximately 50-fold, assuming a typical bulk endosome of 150 nm diameter). Thus, when interpreting pHluorin responses during high-frequency stimulation, CME is rate limiting for most SV cargo, whereas bulk endosome acidification is rate limiting for VAMP4-pHluorin.

The selective capture of VAMP4 by ADBE is not simply due to its high expression at the plasma membrane in comparison to other SV cargo (Raingo et al., 2012). This is because the proportion of plasma membrane VAMP4-pHluorin (23.1% ± 4.0% cerebellar neurons; 36.1% ± 7.0% hippocampal neurons) is comparable to the surface expression of both sybII-pHluorin and Syt1-pHluorin (Gordon et al., 2011; Pan et al., 2015; Zhang et al., 2015), neither of which are selectively accumulated via this endocytosis mode.

We show that a di-leucine sorting motif on VAMP4 is essential for its recovery via ADBE and for ADBE itself. This motif coordinates interactions with adaptor proteins (Peden et al., 2001), suggesting that VAMP4 recruits specific adaptors to mediate both ADBE and its own recovery from the plasma membrane. The cytoplasmic domain of VAMP4 contains other interaction sites for adaptor proteins such as PACS-1 (Hinners et al., 2003), and its SNARE motif can interact with the CALM/AP180 family of monomeric adaptor molecules (Sahlender et al., 2013). It will be important to establish the role of these interactions and how they coordinate the recovery of other ADBE-specific cargoes.

The selective accumulation of VAMP4 during ADBE suggests that SVs formed via this mode of endocytosis may have a specific molecular signature that defines their physiological function in central nerve terminals. It is known that ADBE-derived SVs repopulate the reserve pool, which is only released during intense stimulation after the synchronous release of the readily releasable pool (Richards et al., 2003; Cheung et al., 2010). In agreement we observed a rundown in presynaptic function in VAMP4 knockdown neurons, confirming the requirement for ADBE-derived SVs to maintain neurotransmitter release during intense neuronal activity (Cheung et al., 2010). SV pools have been proposed to have a specific molecular composition that defines their role, in particular the expression of non-canonical forms of sybII (Hua et al., 2011; Raingo et al., 2012; Ramirez et al., 2012). Modulation of VAMP4 expression has bidirectional effects on asynchronous release in neuronal culture (Raingo et al., 2012), suggesting ADBE-derived SVs may replenish this specific functional pool. In support, AP-3b2 knockout mice display large deficits in asynchronous release (Evstratova et al., 2014), agreeing with the essential role for the adaptor protein AP-3 in generating SVs from bulk endosomes (Cheung and Cousin, 2012). Thus, ADBE may produce SVs with a distinct molecular signature that destines them to specifically maintain a SV pool that mediates asynchronous release.

EXPERIMENTAL PROCEDURES

Materials

The pHluorin expression vectors were obtained from the following sources: Syp-pHluorin, Prof. L. Lagnado (University of Sussex); Syt1-pHluorin, Prof. V. Haucke (Leibniz Institute of Molecular Pharmacology); vGLUT1-pHluorin, Prof. R. Edwards (University of California, San Francisco); sybII-pHluorin, Prof. G. Miesenbock (Oxford University); and VAMP4-pHluorin, Prof. Ege Kavalali (UT Southwestern Medical Centre). The sequence encoding VAMP4-pHluorin was cloned into a Clontech EGFP-N1 mammalian expression vector by first removing EGFP and then inserting VAMP4-pHluorin using AgeI and NotI enzymes. The L25A mutant was made via mutagenesis using the primers forward tgaaaggagaaatgctttggaagatgatg; reverse catcatcttccaaagcatttctcc tttca (mutated bases underlined). The empty vector for mCerulean was made as described (Gordon and Cousin, 2013) as were syndapin I and empty mCerulean-tagged pSUPER shRNA vectors (Cheung et al., 2010). Validated shRNA oligonucleotides and their scrambled controls for both CHC (Royle et al., 2005) and VAMP4 (Raingo et al., 2012) were ligated into mCerulean-tagged pSUPER shRNA vectors as described (Cheung et al., 2010). FM1-43 and advasep-7 were from Biotium. VAMP4, synaptophysin, and vGLUT1 antibodies were from Synaptic Systems. Pitstop-2 and syt1 and sybII antibodies were from AbCam. CT92001 was from R&D Systems, and roscovitine was from Merck. Neurobasal media, B-27 supplement, penicillin and streptomycin, minimal essential medium (MEM), and Lipofectamine 2000 were obtained from Invitrogen. The silver enhancement kit was from Nanoprobes, whereas F(ab') 2 fragment anti-rabbit antibodies conjugated to ultrasmall gold particles were from Electron Microscopy Sciences. Osmium tetroxide, paraformaldehyde,

and glutaraldehyde were from Agar Scientific. All other reagents were obtained from Sigma-Aldrich.

Tissue Culture

Primary cultures of cerebellar neurons were prepared from the cerebella of 7-day-old Sprague Dawley rat pups of both sexes (Anggono et al., 2006). Dissociated primary hippocampal neuronal cultures were prepared from E17.5 C56BL/6J mouse embryos of both sexes by trituration of isolated hippocampi to obtain a single cell suspension, which was plated at a density of 5×10^5 cells/coverslip on poly-D-lysine and laminin-coated 25-mm coverslips. Cultures were maintained in neurobasal media supplemented with B-27, 0.5 mM L-glutamine, and 1% v/v penicillin and streptomycin. After 72 hr, cultures were further supplemented with 1 μM cytosine β-d-arabinofuranoside to inhibit glial proliferation.

Transfections

Cerebellar neurons were transfected between 5 and 7 days in culture, whereas hippocampal neurons were transfected between 6 and 8 days in culture with Lipofectamine 2000 (Gordon et al., 2011). In most experiments, two constructs were co-expressed; pHluorin vectors were cotransfected with either mCerulean empty vector or mCerulean expressing shRNA vectors. Both sybII-pHluorin and syp-pHluorin were expressed in the absence of other vectors unless specifically stated in the legends. Cerebellar neurons were imaged after 8–10 days in culture, whereas hippocampal neurons were imaged after 13–16 days.

Imaging of pHluorin Responses

Cerebellar neuron cultures were removed from culture medium and left for 10 min in incubation medium (170 mM NaCl, 3.5 mM KCl, 0.4 mM KH_2PO_4, 20 mM TES (*N*-tris[hydroxy-methyl]-methyl-2-aminoethane-sulphonic acid), 5 mM $NaHCO_3$, 5 mM glucose, 1.2 mM Na_2SO_4, 1.2 mM $MgCl_2$, and 1.3 mM $CaCl_2$ [pH 7.4]). They were mounted in a Warner imaging chamber with embedded parallel platinum wires (RC-21BRFS) and placed on the stage of a Zeiss Axio Observer A1 epifluorescence microscope. Transfected neurons were visualized with a Zeiss Plan Apochromat ×40 oil immersion objective (NA 1.3) at 430 nm excitation (to illuminate mCer), whereas pHluorin reporters were visualized at 500 nm (both using a dichroic > 525 nm and long-pass emission filter > 535 nm). Cultures were subjected to continuous perfusion with incubation medium and stimulated with a train of either 400 action potentials delivered at 40 Hz (100 mA, 1-ms pulse width) or 300 action potentials delivered at 10 Hz where indicated. At the end of the experiment, cultures were challenged with alkaline imaging buffer (50 mM NH_4Cl substituted for 50 mM NaCl) to reveal total pHluorin fluorescence. Where indicated cultures were also challenged with acidic imaging buffer (20 mM MES substituted for 20 mM TES [pH 5.5]). Fluorescent images were captured at 4-s intervals using a Zeiss AxioCam MRm Rev.3 digital camera and processed offline using Image J 1.43 software. Regions of interest of identical size were placed over nerve terminals, and the total fluorescence intensity was monitored over time. Only regions that responded to action potential stimulation were selected for analysis. All statistical analyses were performed using Microsoft Excel and GraphPad Prism software. Where required, traces were decay corrected using a mono-exponential decay function fitted to the first 15 points of acquisition. The pHluorin fluorescence change was calculated as $F\Delta/F_0$, and n refers to the number of individual coverslips examined.

Imaging of hippocampal cultures was performed in essentially the same manner apart from perfusion with an altered imaging buffer (136 mM NaCl, 2.5 mM KCl, 2 mM $CaCl_2$, 1.3 mM $MgCl_2$, 10 mM glucose, and 10 mM HEPES [pH 7.4] supplemented with 10 μM 6-cyano-7-nitroquinoxaline-2,3-dione and 50 μM DL-2-Amino-5-phosphonopentanoic acid).

Dextran Uptake

The uptake of TMR-dextran (40 kDa) was monitored as described previously (Clayton et al., 2008). Briefly, cerebellar neurons were removed from culture medium, left for 10 min in incubation medium, and then stimulated with a train of 400 action potentials (40 Hz, 10 s). TMR-dextran (50 μM) was present during the stimulus and was washed away immediately after stimulation. The extent of loading was determined by the number of fluorescent puncta in a defined field of view (130 × 130 μm) using a 40× oil immersion objective at 550 nm excitation and >575 nm emission. Thresholding analysis was performed to discount regions too large to represent individual nerve terminals (diameter greater than 2 μm). The average number of dextran puncta per field for each experiment (usually eight fields of view per experiment) were averaged for the same conditions and subtracted from background fluorescence. The final value for dextran puncta was obtained by averaging the individual averages from at least three independent experiments (n is taken as the number of experiments). To ensure the density of nerve terminals was consistent between fields and experimental conditions, experiments were always performed on the same set of cultures. Cultures were used between 8 and 10 days in vitro. Experiments with hippocampal neurons were performed in an almost identical manner, with the exception of the 10-min repolarization in altered imaging buffer. Hippocampal neurons were used between 14 and 16 days in vitro.

Experiments using neurons transfected with either shRNA or overexpression vectors were performed in the same manner, with the number of dextran puncta per μm of axon calculated and then normalized to control values. In both cases n is the number neurons analyzed from at least three independent coverslips.

Immunofluorescence

Immunolabeling was performed as described (Gordon et al., 2011). Briefly transfected neurons were visualized at 480 nm after incubation with anti-GFP antibodies to enhance the signal from mCerulean expressing neurons. Endogenous CHC or VAMP4 were visualized at 550 nm (antibody dilutions were 1:250 and 1:500 for CHC and VAMP4, respectively). Identically sized regions of interest were placed over transfected neurons in the same field of view, along with background regions. The level of either CHC or VAMP4 expression was calculated by subtracting background autofluorescence prior to calculating the ratio for transfected/non-transfected expression levels.

Immunoelectron Microscopy

Cerebellar neurons were removed from culture medium, left for 10 min in incubation medium, and then either stimulated with a train of 400 action potentials (40 Hz, 10 s) or left to rest. Cultures were immediately fixed in 2% paraformaldehyde/0.5% glutaraldehyde in 0.1 M sodium phosphate buffer (PB [pH 7.4]) at room temperature. After three washes in 0.1 M PB, cerebellar neurons were permeabilized using 2% BSA/0.1% Tx-100 in 0.1 M PB for 1 hr. Cultures were then incubated with VAMP4 antibody (1:100) in 2% BSA/0.1% Tx-100 in 0.1 M PB for 1 hr. After three washes in 2% BSA in 0.1 M PB, cerebellar neurons were incubated with ultrasmall gold anti-rabbit conjugated Fab'antibodies for 1 hr in 2% BSA in 0.1 M PB. Cells were washed three times in 0.1 M PB and post-fixed in 2% glutaraldehyde in 0.1 M PB for 30 min. After three washes in 0.1 M PB, cultures were subjected to HQ Silver Enhancement as per the manufacturer's instructions (Nanoprobes). After further washes in dH_2O and then 0.1 M PB, cerebellar neurons were stained using 1% osmium tetroxide in 0.1 M PB for 30 min. After washing, cultures were subjected to post hoc staining with uranyl acetate before dehydration and embedding using Durcupan resin. Samples were sectioned at 70- to 900-nm thickness and collected on formvar-coated slot grids (Agar Scientific). Grids were stained with lead citrate before being viewed on a JEOL-1200 EX transmission electron microscope.

Bulk Endosome Enrichment

Cerebellar neuron cultures were left to repolarize in incubation medium for 1 hr to minimize existing bulk endosomes and were then stimulated for 2 min with 50 mM KCl in the presence of 10 μM FM1-43. Cells were washed once with incubation medium and then twice with incubation medium supplemented with 200 nM Advasep-7. Cerebellar neurons were collected in buffer containing 250 mM sucrose and 3 mM imidazole [pH 7.4] and mechanically broken using a ball-bearing cell cracker (European Molecular Biology Laboratory), with the lysate spun for 15 min at 1,200 g. The post nuclear supernatant was deposited at the base of a discontinuous Nycodenz (Axis-Shield) gradient (12%, 20%, 25%, and 35% in 3 mM imidazole, 0.5 mM EDTA [pH 7.4]). The samples were centrifuged for 90 min at 170,000 g in an Optima MAX-XP Tabletop Ultracentrifuge (Beckman Coulter). The different fractions were collected as indicated in Figure 6A, with bulk endosomes found at the interface between

12% and 20% Nycodenz and SVs between 20% and 25% Nycodenz. The fluorescence of all fractions was monitored in a TD-700 fluorometer (Turner Designs) to reveal the presence of labeled compartments.

For protein biochemistry studies, stimulated fractions 2 (bulk endosome) and 5 (SVs) were lysed in SDS sample buffer (67 mM SDS, 2 mM EGTA, 9.3% glycerol, 12% β-mercaptoethanol, bromophenol blue, and 67 mM Tris). Samples were resolved on SDS-PAGE and transferred onto nitrocellulose membranes for western blotting. Primary antibodies were used at the following dilutions: VAMP4, 1:2,000; syt1, 1:1500; syp, 1:8,000; sybII, 1:8,000; vGLUT, 1:1,500. These antibodies were amplified using IRDye anti-mouse, rabbit, and guinea pig secondary antibodies (all diluted at 1:10,000; LI-COR Biosciences). Membranes were imaged using an Odyssey 9120 Infrared Imaging System (LI-COR Biosciences) and analyzed using Image Studio Lite (LI-COR Biosciences). The intensity of the endosome and SVs fractions from the same membrane were calculated and expressed as an abundance ratio (endosome/SV) after normalizing to total protein content.

Cerebellar neuron fractions were processed in an identical manner for electron microscopy except for the fact that HRP (10 mg/ml) was used instead of FM1-43 and Advasep-7 was omitted from the wash step. The bulk endosome and SV samples were fixed in 2% glutaraldehyde for 30 min at 37°C and washed in 100 mM Tris (pH 7.4). Samples were incubated in 0.1% diaminobenzidine and 0.2% H_2O_2 until color developed. The samples were stained with 1% osmium tetroxide and dehydrated using ethanol and polypropylene oxide and embedded using Durcupan resin. Samples were sectioned, mounted on grids, and viewed using a FEI Tecnai 12 transmission electron microscope. HRP-labeled structures were identified and their diameter was calculated by taking the average of the longest and shortest diameters of individual endosomes using ImageJ (NIH). A cutoff of 80 nm was used to separate bulk endosomes from SVs (30–60 nm).

Statistical Analysis

A Student's t test was performed for comparisons between two datasets. For greater than two datasets, a one-way ANOVA was employed. For comparisons between fluorescence responses over time, or where greater than one variable was being compared, a two-way ANOVA was performed.

SUPPLEMENTAL INFORMATION

Supplemental Information includes eight figures and can be found with this article online at http://dx.doi.org/10.1016/j.neuron.2015.10.043.

AUTHOR CONTRIBUTIONS

Conceptualization, M.A.C.; Methodology, M.A.C., K.J.S., J.C.N.-F., T.H.G., and A.C.K.; Formal analysis, J.C.N.-F. and A.C.K.; Investigation, J.C.N.-F., T.H.G., A.C.K., and K.J.S.; Writing – original draft, M.A.C., K.J.S., J.C.N.-F., and A.C.K.; Supervision, M.A.C. and K.J.S.; Funding acquisition, M.A.C.

ACKNOWLEDGMENTS

This work was supported by the Medical Research Council (grant number G1002117), a Marie-Curie Initial Training Network grant (project number 289581: *NPlast*), and a BBSRC PhD studentship. We thank John James and Martin Kierans (University of Dundee), Fiona Young (Western General Hospital, Edinburgh), and Stephen Mitchell (University of Edinburgh) for expert technical assistance.

Received: June 5, 2015
Revised: September 22, 2015
Accepted: October 15, 2015
Published: November 19, 2015

REFERENCES

Anggono, V., Smillie, K.J., Graham, M.E., Valova, V.A., Cousin, M.A., and Robinson, P.J. (2006). Syndapin I is the phosphorylation-regulated dynamin I partner in synaptic vesicle endocytosis. Nat. Neurosci. *9*, 752–760.

Atluri, P.P., and Ryan, T.A. (2006). The kinetics of synaptic vesicle reacidification at hippocampal nerve terminals. J. Neurosci. *26*, 2313–2320.

Bal, M., Leitz, J., Reese, A.L., Ramirez, D.M., Durakoglugil, M., Herz, J., Monteggia, L.M., and Kavalali, E.T. (2013). Reelin mobilizes a VAMP7-dependent synaptic vesicle pool and selectively augments spontaneous neurotransmission. Neuron *80*, 934–946.

Barysch, S.V., Jahn, R., and Rizzoli, S.O. (2010). A fluorescence-based in vitro assay for investigating early endosome dynamics. Nat. Protoc. *5*, 1127–1137.

Burgoyne, R.D., and Cambray-Deakin, M.A. (1988). The cellular neurobiology of neuronal development: the cerebellar granule cell. Brain Res. *472*, 77–101.

Cheung, G., and Cousin, M.A. (2012). Adaptor protein complexes 1 and 3 are essential for generation of synaptic vesicles from activity-dependent bulk endosomes. J. Neurosci. *32*, 6014–6023.

Cheung, G., Jupp, O.J., and Cousin, M.A. (2010). Activity-dependent bulk endocytosis and clathrin-dependent endocytosis replenish specific synaptic vesicle pools in central nerve terminals. J. Neurosci. *30*, 8151–8161.

Clayton, E.L., and Cousin, M.A. (2009a). The molecular physiology of activity-dependent bulk endocytosis of synaptic vesicles. J. Neurochem. *111*, 901–914.

Clayton, E.L., and Cousin, M.A. (2009b). Quantitative monitoring of activity-dependent bulk endocytosis of synaptic vesicle membrane by fluorescent dextran imaging. J. Neurosci. Methods *185*, 76–81.

Clayton, E.L., Evans, G.J., and Cousin, M.A. (2008). Bulk synaptic vesicle endocytosis is rapidly triggered during strong stimulation. J. Neurosci. *28*, 6627–6632.

Clayton, E.L., Anggono, V., Smillie, K.J., Chau, N., Robinson, P.J., and Cousin, M.A. (2009). The phospho-dependent dynamin-syndapin interaction triggers activity-dependent bulk endocytosis of synaptic vesicles. J. Neurosci. *29*, 7706–7717.

Clayton, E.L., Sue, N., Smillie, K.J., O'Leary, T., Bache, N., Cheung, G., Cole, A.R., Wyllie, D.J., Sutherland, C., Robinson, P.J., and Cousin, M.A. (2010). Dynamin I phosphorylation by GSK3 controls activity-dependent bulk endocytosis of synaptic vesicles. Nat. Neurosci. *13*, 845–851.

Diril, M.K., Wienisch, M., Jung, N., Klingauf, J., and Haucke, V. (2006). Stonin 2 is an AP-2-dependent endocytic sorting adaptor for synaptotagmin internalization and recycling. Dev. Cell *10*, 233–244.

Egashira, Y., Takase, M., and Takamori, S. (2015). Monitoring of vacuolar-type H+ ATPase-mediated proton influx into synaptic vesicles. J. Neurosci. *35*, 3701–3710.

Evans, G.J., and Cousin, M.A. (2007). Activity-dependent control of slow synaptic vesicle endocytosis by cyclin-dependent kinase 5. J. Neurosci. *27*, 401–411.

Evstratova, A., Chamberland, S., Faundez, V., and Tóth, K. (2014). Vesicles derived via AP-3-dependent recycling contribute to asynchronous release and influence information transfer. Nat. Commun. *5*, 5530.

Gordon, S.L., and Cousin, M.A. (2013). X-linked intellectual disability-associated mutations in synaptophysin disrupt synaptobrevin II retrieval. J. Neurosci. *33*, 13695–13700.

Gordon, S.L., Leube, R.E., and Cousin, M.A. (2011). Synaptophysin is required for synaptobrevin retrieval during synaptic vesicle endocytosis. J. Neurosci. *31*, 14032–14036.

Granseth, B., Odermatt, B., Royle, S.J., and Lagnado, L. (2006). Clathrin-mediated endocytosis is the dominant mechanism of vesicle retrieval at hippocampal synapses. Neuron *51*, 773–786.

Heerssen, H., Fetter, R.D., and Davis, G.W. (2008). Clathrin dependence of synaptic-vesicle formation at the Drosophila neuromuscular junction. Curr. Biol. *18*, 401–409.

Hinners, I., Wendler, F., Fei, H., Thomas, L., Thomas, G., and Tooze, S.A. (2003). AP-1 recruitment to VAMP4 is modulated by phosphorylation-dependent binding of PACS-1. EMBO Rep. *4*, 1182–1189.

Hua, Z., Leal-Ortiz, S., Foss, S.M., Waites, C.L., Garner, C.C., Voglmaier, S.M., and Edwards, R.H. (2011). v-SNARE composition distinguishes synaptic vesicle pools. Neuron *71*, 474–487.

Hua, Y., Woehler, A., Kahms, M., Haucke, V., Neher, E., and Klingauf, J. (2013). Blocking endocytosis enhances short-term synaptic depression under conditions of normal availability of vesicles. Neuron *80*, 343–349.

Kasprowicz, J., Kuenen, S., Miskiewicz, K., Habets, R.L., Smitz, L., and Verstreken, P. (2008). Inactivation of clathrin heavy chain inhibits synaptic recycling but allows bulk membrane uptake. J. Cell Biol. *182*, 1007–1016.

Kavalali, E.T., and Jorgensen, E.M. (2014). Visualizing presynaptic function. Nat. Neurosci. *17*, 10–16.

Kelly, B.T., and Owen, D.J. (2011). Endocytic sorting of transmembrane protein cargo. Curr. Opin. Cell Biol. *23*, 404–412.

Kokotos, A.C., and Cousin, M.A. (2015). Synaptic vesicle generation from central nerve terminal endosomes. Traffic *16*, 229–240.

Kononenko, N.L., Puchkov, D., Classen, G.A., Walter, A.M., Pechstein, A., Sawade, L., Kaempf, N., Trimbuch, T., Lorenz, D., Rosenmund, C., et al. (2014). Clathrin/AP-2 mediate synaptic vesicle reformation from endosome-like vacuoles but are not essential for membrane retrieval at central synapses. Neuron *82*, 981–988.

Koo, S.J., Markovic, S., Puchkov, D., Mahrenholz, C.C., Beceren-Braun, F., Maritzen, T., Dernedde, J., Volkmer, R., Oschkinat, H., and Haucke, V. (2011). SNARE motif-mediated sorting of synaptobrevin by the endocytic adaptors clathrin assembly lymphoid myeloid leukemia (CALM) and AP180 at synapses. Proc. Natl. Acad. Sci. USA *108*, 13540–13545.

López-Murcia, F.J., Royle, S.J., and Llobet, A. (2014). Presynaptic clathrin levels are a limiting factor for synaptic transmission. J. Neurosci. *34*, 8618–8629.

Pan, P.Y., Marrs, J., and Ryan, T.A. (2015). Vesicular Glutamate Transporter 1 orchestrates recruitment of other synaptic vesicle cargo proteins during synaptic vesicle recycling. J. Biol. Chem. *290*, 22593–22601.

Peden, A.A., Park, G.Y., and Scheller, R.H. (2001). The Di-leucine motif of vesicle-associated membrane protein 4 is required for its localization and AP-1 binding. J. Biol. Chem. *276*, 49183–49187.

Raingo, J., Khvotchev, M., Liu, P., Darios, F., Li, Y.C., Ramirez, D.M., Adachi, M., Lemieux, P., Toth, K., Davletov, B., and Kavalali, E.T. (2012). VAMP4 directs synaptic vesicles to a pool that selectively maintains asynchronous neurotransmission. Nat. Neurosci. *15*, 738–745.

Ramirez, D.M., Khvotchev, M., Trauterman, B., and Kavalali, E.T. (2012). Vti1a identifies a vesicle pool that preferentially recycles at rest and maintains spontaneous neurotransmission. Neuron *73*, 121–134.

Rao, Y., Rückert, C., Saenger, W., and Haucke, V. (2012). The early steps of endocytosis: from cargo selection to membrane deformation. Eur. J. Cell Biol. *91*, 226–233.

Richards, D.A., Guatimosim, C., Rizzoli, S.O., and Betz, W.J. (2003). Synaptic vesicle pools at the frog neuromuscular junction. Neuron *39*, 529–541.

Royle, S.J., Bright, N.A., and Lagnado, L. (2005). Clathrin is required for the function of the mitotic spindle. Nature *434*, 1152–1157.

Sahlender, D.A., Kozik, P., Miller, S.E., Peden, A.A., and Robinson, M.S. (2013). Uncoupling the functions of CALM in VAMP sorting and clathrin-coated pit formation. PLoS ONE *8*, e64514.

Sankaranarayanan, S., and Ryan, T.A. (2000). Real-time measurements of vesicle-SNARE recycling in synapses of the central nervous system. Nat. Cell Biol. *2*, 197–204.

von Kleist, L., Stahlschmidt, W., Bulut, H., Gromova, K., Puchkov, D., Robertson, M.J., MacGregor, K.A., Tomilin, N., Pechstein, A., Chau, N., et al. (2011). Role of the clathrin terminal domain in regulating coated pit dynamics revealed by small molecule inhibition. Cell *146*, 471–484.

Watanabe, S., Rost, B.R., Camacho-Pérez, M., Davis, M.W., Söhl-Kielczynski, B., Rosenmund, C., and Jorgensen, E.M. (2013). Ultrafast endocytosis at mouse hippocampal synapses. Nature *504*, 242–247.

Watanabe, S., Trimbuch, T., Camacho-Pérez, M., Rost, B.R., Brokowski, B., Söhl-Kielczynski, B., Felies, A., Davis, M.W., Rosenmund, C., and Jorgensen, E.M. (2014). Clathrin regenerates synaptic vesicles from endosomes. Nature *515*, 228–233.

Zhang, N., Gordon, S.L., Fritsch, M.J., Esoof, N., Campbell, D.G., Gourlay, R., Velupillai, S., Macartney, T., Peggie, M., van Aalten, D.M., et al. (2015). Phosphorylation of synaptic vesicle protein 2A at Thr84 by casein kinase 1 family kinases controls the specific retrieval of synaptotagmin-1. J. Neurosci. *35*, 2492–2507.

Neuron

Supplemental Information

VAMP4 Is an Essential Cargo Molecule for Activity-Dependent Bulk Endocytosis

Jessica C. Nicholson-Fish, Alexandros C. Kokotos, Thomas H. Gillingwater, Karen J. Smillie, and Michael A. Cousin

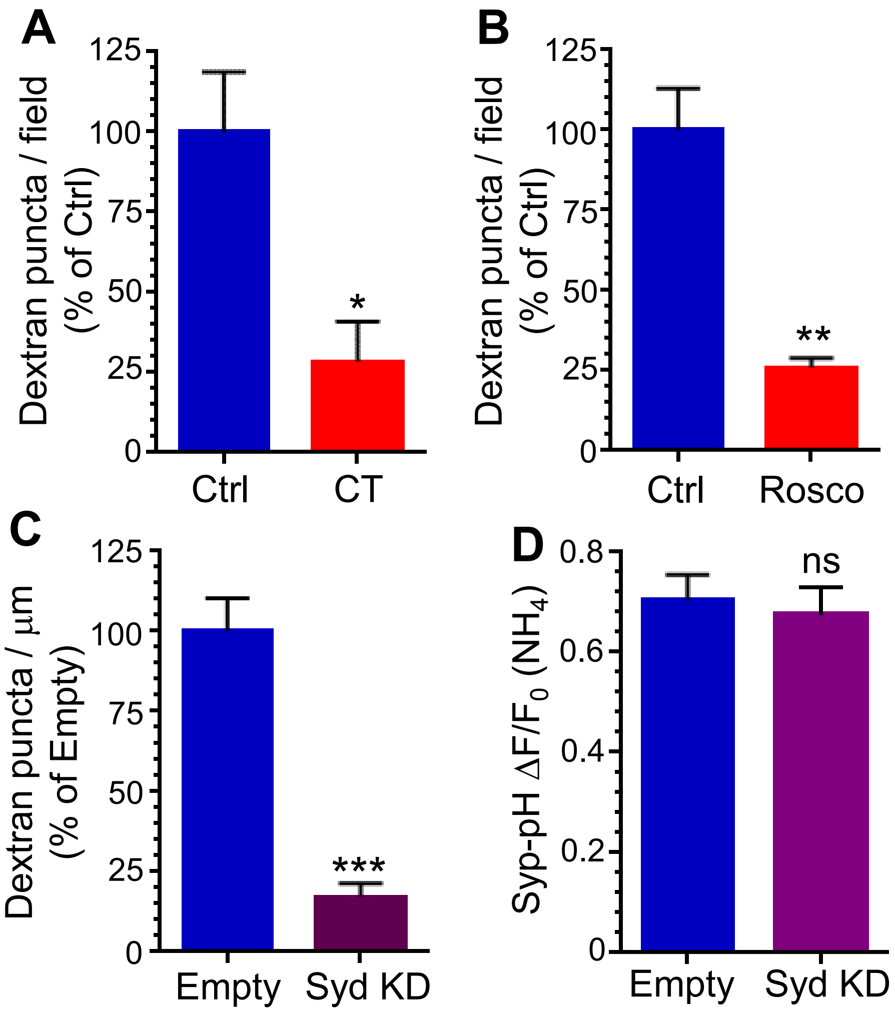

Figure S1 – *Related to Figure 2: Inhibition of ADBE arrests TMR-dextran uptake.* **A,B**) Cerebellar neurons were stimulated with two sequential action potential trains (S1 and S2) 10 minutes apart (both 40 Hz 10 s). Cultures were incubated with either **A)** 2 µM CT99021 (CT) or **B)** 10 µM roscovitine (Ros) 10 minutes before S1. At S2 50 µM TMR-dextran was added and immediately washed away on termination of stimulation. Quantification of dextran puncta per field ± SEM normalised to control is displayed (n = 4 Ctrl, n = 5 CT, n = 3 for Ctrl and Ros, ** = $p<0.01$, * = $p<0.05$, students t test). **C**) Cerebellar neurons were transfected with either empty shRNA vector or shRNA against syndapin I (Syd KD). Cultures were stimulated at 40 Hz for 10 s in the presence of 50 µM TMR-dextran. Quantification of dextran puncta per µm ± SEM normalised to empty control (n = 33 Empty, n = 40 Syd KD, *** = $p<0.01$, students t test). **D**) Cerebellar neurons transfected with synaptophysin-pHluorin (syp-pH) and either empty shRNA (empty) or shRNA against syndapin I (Syd KD) were stimulated with a train of 400 action potentials (40 Hz). Quantification of the evoked syp-pH response $\Delta F/F_0$ ± SEM normalised to the total pHluorin pool (NH_4) for both empty (blue) and Syd KD (purple), n = 8 Empty, n = 5 Syd KD, ns, students t test.

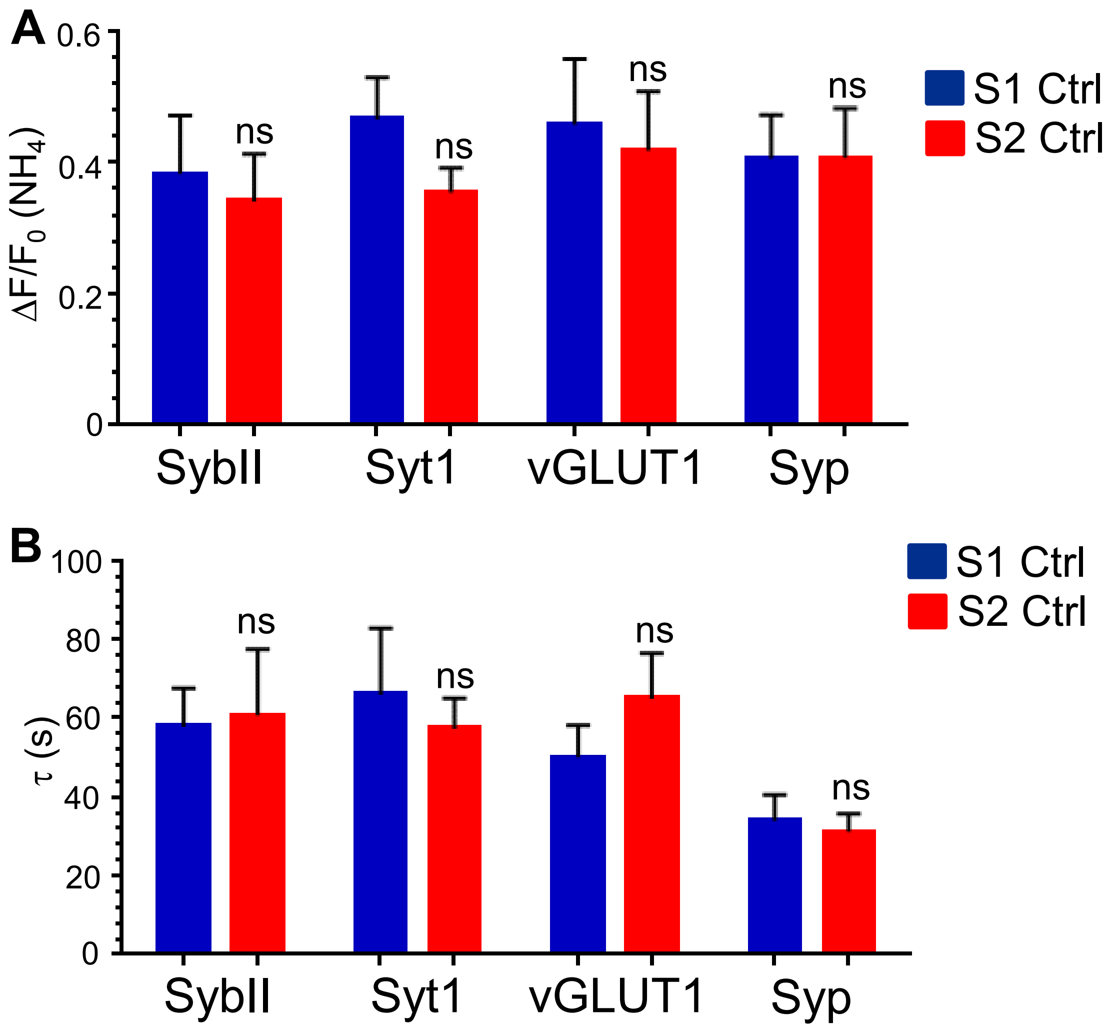

Figure S2 – *Related to Figure 4: Evoked response of multiple pHluorin reporters is reproducible during intense stimulation.* Cerebellar neurons transfected with the pHluorin reporters synaptobrevinII-pHluorin (SybII), synaptotagmin-1-pHluorin (Syt1), vGLUT1-pHluorin (vGLUT1) or synaptophysin-pHluorin (Syp) were stimulated with two sequential action potential trains (S1 and S2) 10 minutes apart (both 40 Hz 10 s). **A**) Quantification of the evoked pHluorin peak response ($\Delta F/F_0 \pm$ SEM) normalised to the total pHluorin pool (NH_4) for both S1 (blue) and S2 (red) traces. **B**) Quantification of the average time constant (τ) ± SEM for both S1 (blue) and S2 (red) traces (n = 4 SybII, n = 10 Syt1, n = 5 vGLUT1, n = 6 Syp, ns students t test).

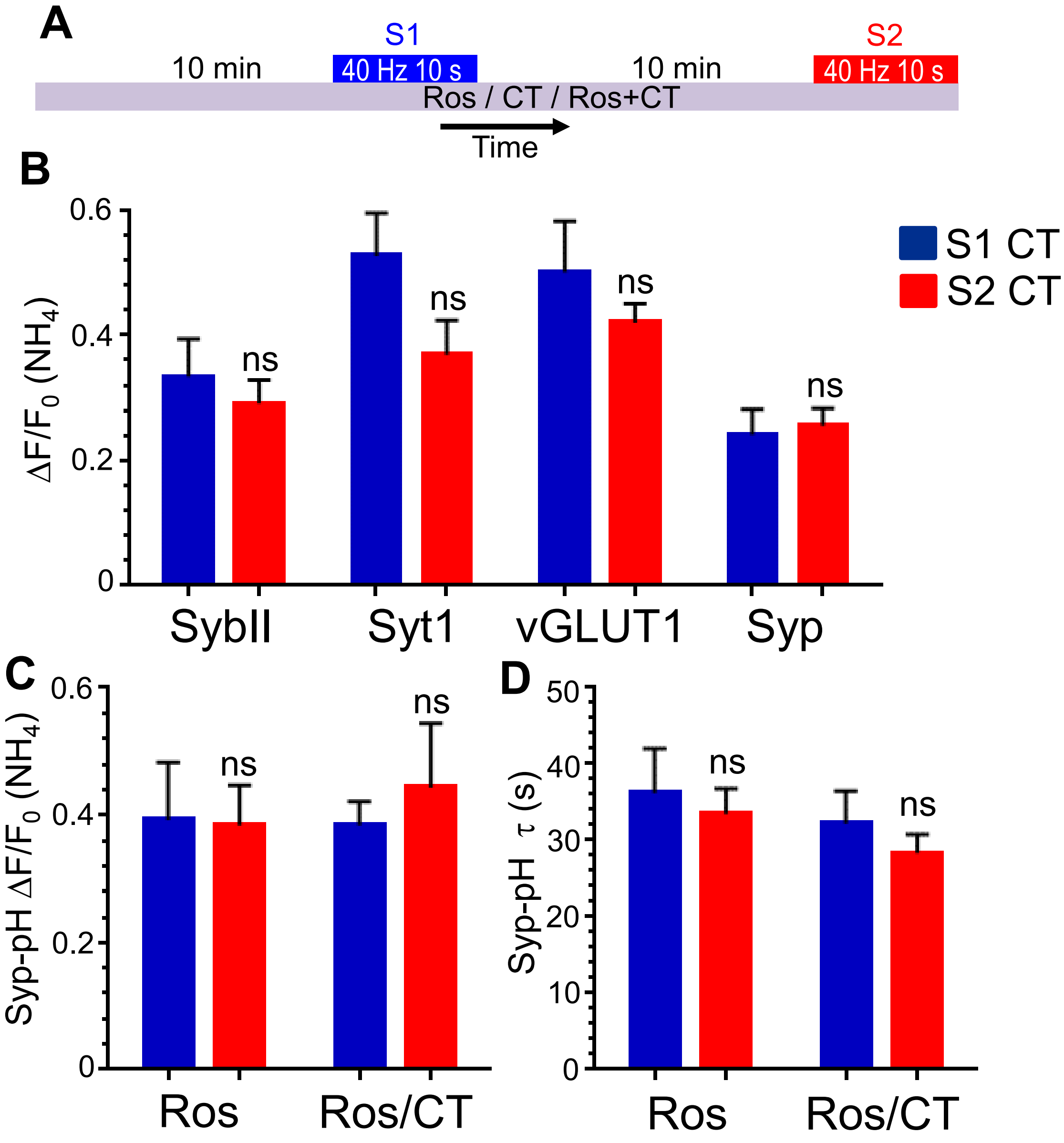

Figure S3 – *Related to Figure 4: Inhibition of ADBE does not affect the extent of response for multiple pHluorin reporters.* **A)** Cerebellar neurons transfected with the pHluorin reporters synaptobrevinII-pHluorin (SybII), synaptotagmin-1-pHluorin (Syt1), vGLUT1-pHluorin (vGLUT1) or synaptophysin-pHluorin (Syp) were stimulated with two sequential action potential trains (S1 and S2) 10 minutes apart (both 40 Hz 10 s). Cultures were incubated with either 2 µM CT99021 (CT), 10 µM roscovitine (Ros) or both 10 minutes prior to S1 and then continuously onwards. **B**) Quantification of the evoked pHluorin response $\Delta F/F_0$ ± SEM normalised to the total pHluorin pool (NH_4) for SybII, Syt1, vGLUT1 and Syp response for both S1 (blue) and S2 (red) traces in the presence of CT (n = 4 SybII, n = 10 Syt1, n = 5 vGLUT1, n = 6 Syp, ns students t test). **C**) Quantification of the evoked Syp-pH response $\Delta F/F_0$ ± SEM normalised to the total pHluorin pool (NH_4) or **D**) average time constant (τ) ± SEM for both S1 (blue) and S2 (red) in the presence of either Ros or both CT and Ros (n = 5 Ros, n = 4 Ros/CT, ns students t test)).

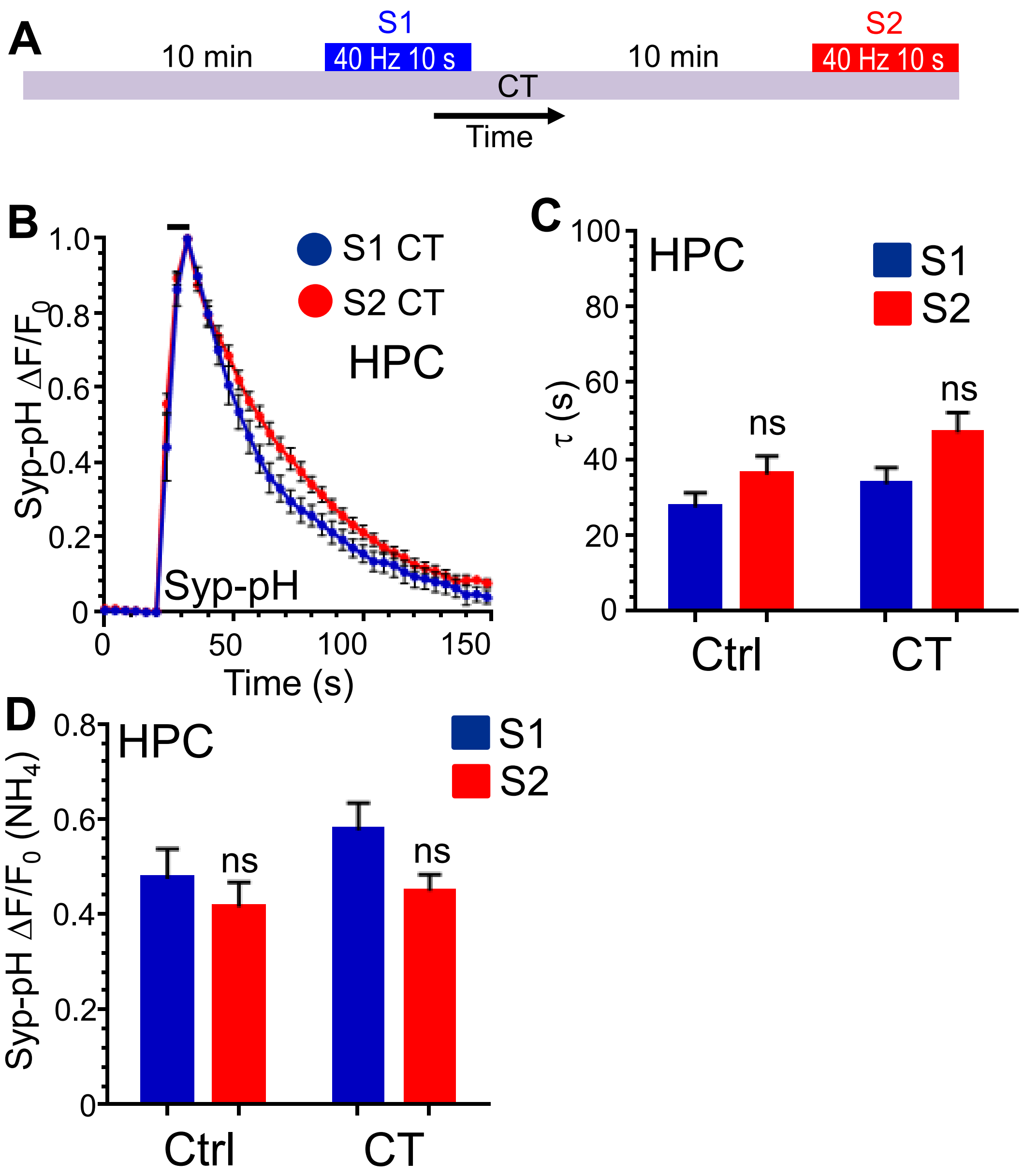

Figure S4 – *Related to results section paragraph 9: Synaptophysin-pHluorin is not retrieved by ADBE in hippocampal neurons.* **A**) Hippocampal (HPC) neurons transfected with synaptophysin-pHluorin (syp-pH) were stimulated with two sequential action potential trains (S1 and S2) 10 minutes apart (both 40 Hz 10 s). Cultures were incubated with 2 μM CT99021 (CT) 10 minutes prior to S1 and then continuously onwards where indicated. **B**) Average time course of the syp-pH fluorescent response in the presence of CT presented as $\Delta F/F_0$ ± SEM at both S1 (blue) and S2 (red). Bar indicates period of stimulation. **C**) Quantification of the average time constant (τ) ± SEM for both S1 (blue) and S2 (red). **D**) Quantification of the evoked syp-pH response $\Delta F/F_0$ ± SEM normalised to the total pHluorin pool (NH_4) for both S1 (blue) and S2 (red), n = 4 Ctrl, n = 5 CT, ns, one-way ANOVA.

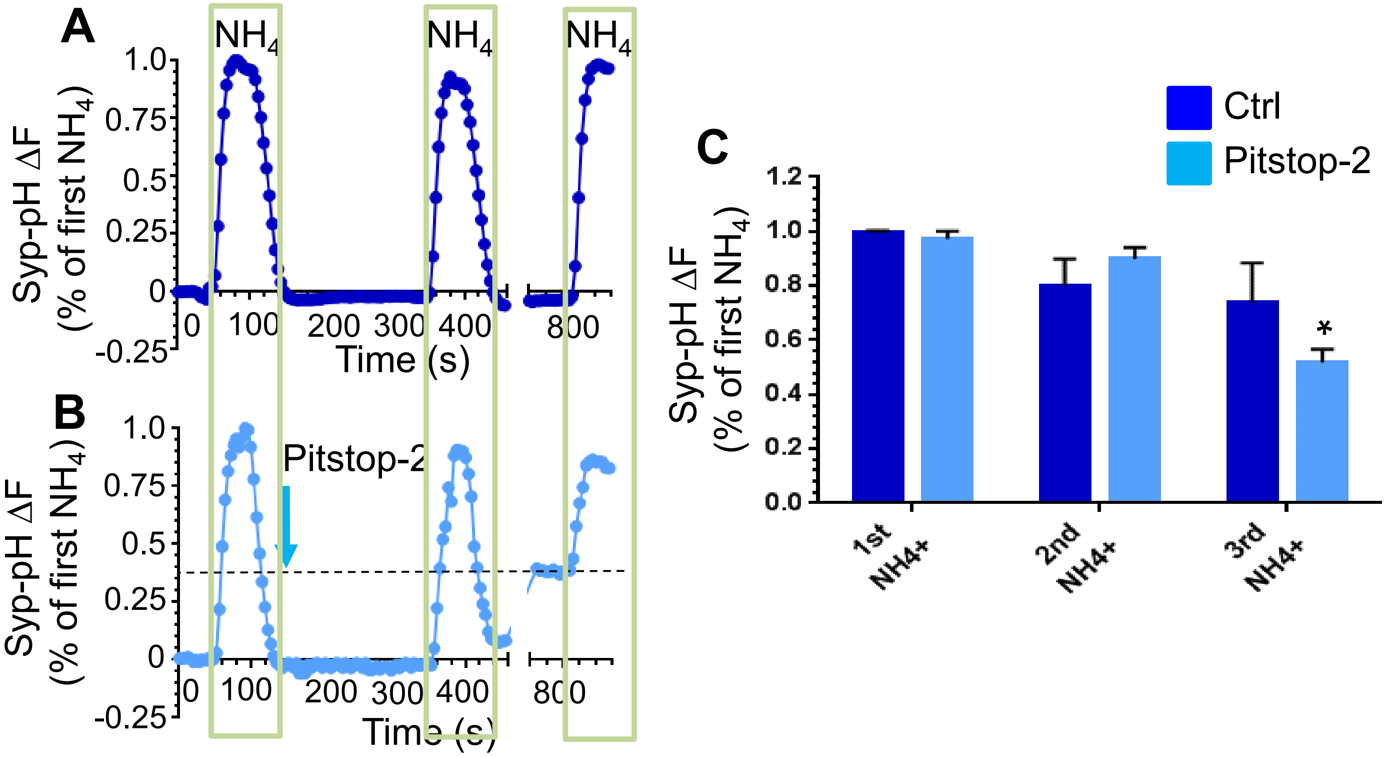

Figure S5 – *Related to Figure 5: Pitstop-2 only affects SV acidification after 10 minutes.* **A)** Cerebellar neurons transfected with synaptophysin-pHluorin (syp-pH) were challenged with a pulse of ammonium buffer (NH_4) to determine the maximum dynamic range of the fluorescent response (indicated by shaded area). Neurons were subsequently challenged with ammonium buffer after a further 4 minutes and 10 minutes. **B)** Neurons were subjected to an identical protocol apart from incubation with 15 µM pitstop-2 immediately after the first alkaline challenge (arrow). **C)** Dynamic range of the syp-pH response with (light blue) and without (dark blue) pitstop-2 is presented ± SEM (normalised to the first ammonia pulse, n = 3 for both, * = $p<0.05$, one-way ANOVA). Pitstop-2 reduces the syp-pH dynamic range after 10 mins, which is not within the time course of the experiments performed in this manuscript.

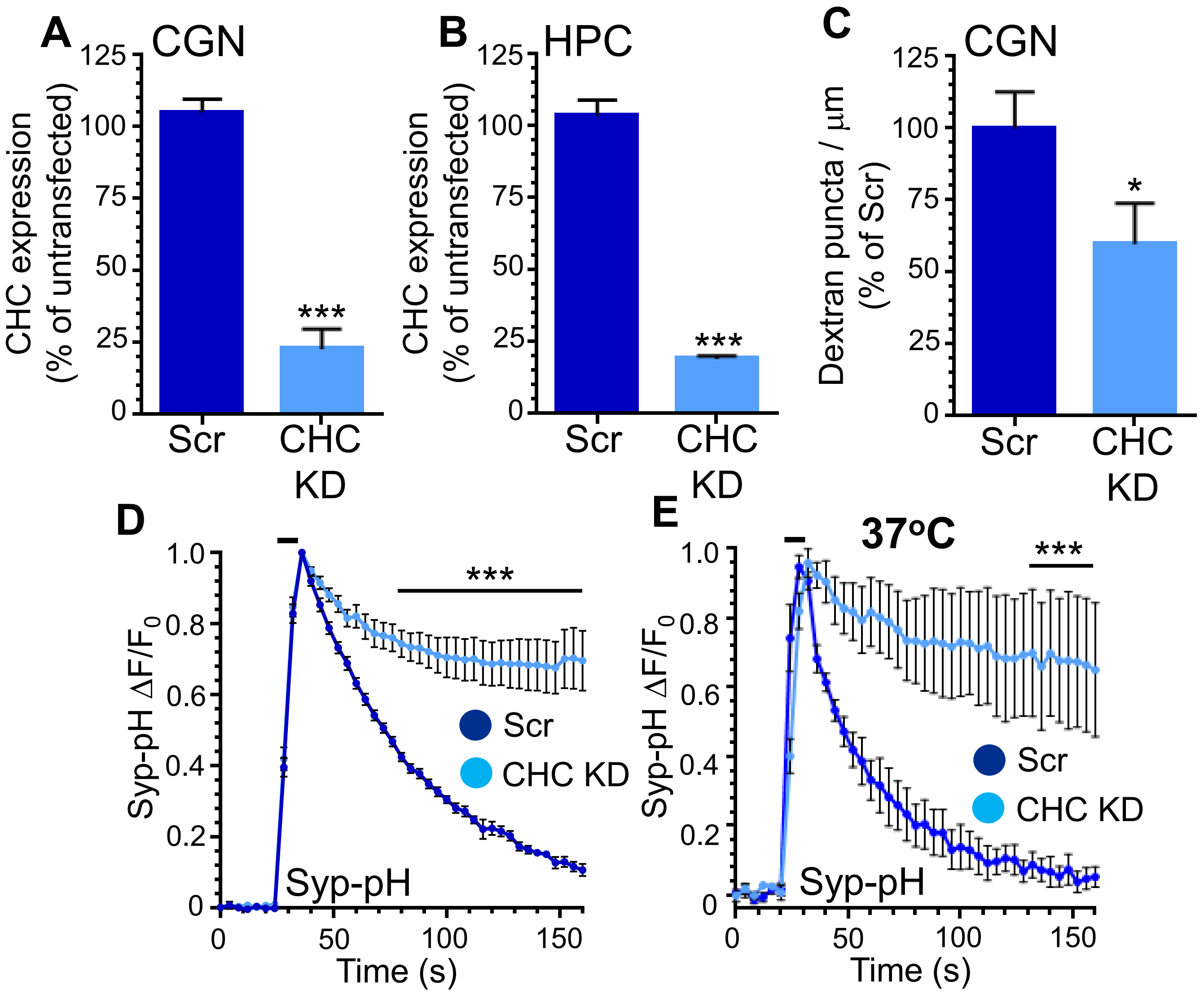

Figure S6 – *Related to Figure 5: Inhibition of CME arrests syp-pHluorin retrieval during intense stimulation.* **A,B)** Cerebellar (CGN) or hippocampal (HPC) neurons were transfected with either scrambled shRNA (Scr) or clathrin heavy chain (CHC) shRNA (CHC KD). The extent of CHC knockdown was quantified by immunofluorescence and presented as a percentage of untransfected neurons in the same field of view ± SEM (CGN, n=16 Scr, n=12 CHC KD, HPC, n=16 Scr, n=15, CHC KD, *** = p<0.001 students t test). **C)** CGNs transfected with either Scr or CHC KD shRNA were stimulated with an action potential train (40 Hz 10 s) in the presence of 50 mM TMR-dextran. Quantification of dextran puncta per µm ± SEM normalised to Scr control (n = 28 Scr, n = 30 CHC, * = p<0.05, students t test). **D,E)** CGNs or HPCs transfected with synaptophysin-pHluorin (syp-pH) and either Scr or CHC KD shRNA were stimulated (40 Hz 10 s) as indicated by bar. Average time course $\Delta F/F_0$ ± SEM of the evoked syp-pH response is displayed (CGN, n = 7 Scr, n = 8 CHC KD, HPC, n = 5 Scr, n = 4 CHC KD *** = p<0.001, two-way ANOVA). In **(E)** experiments were performed at physiological temperature (37°C).

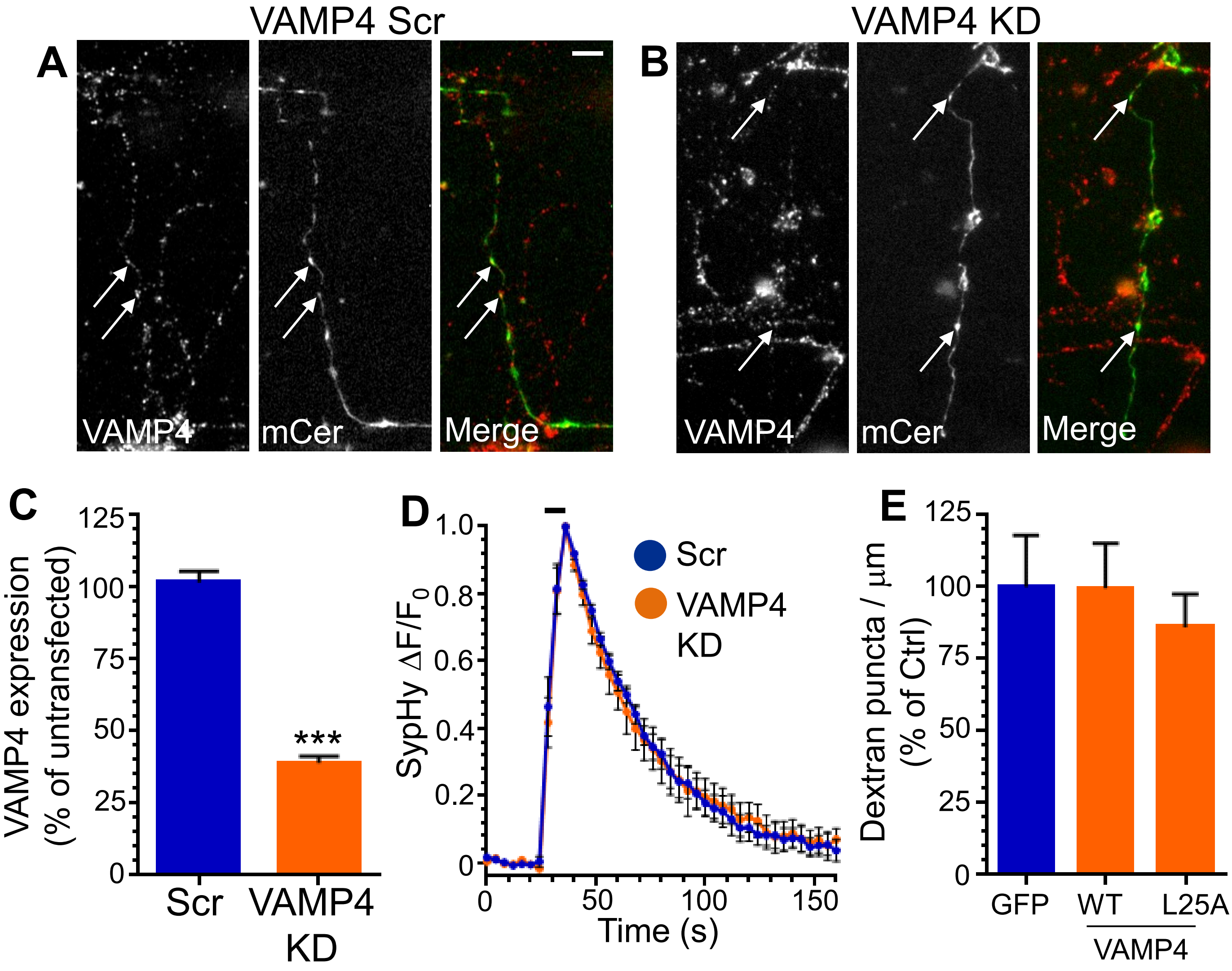

Supplementary Figure 7 – *Related to Figures 7 & 8: VAMP4 is essential for ADBE*. **A,B)** Immunofluorescence images show cerebellar neurons (CGNs) transfected with either scrambled (Scr, **A**) or VAMP4 shRNA (VAMP4 KD, **B**). Images display transfected neuron (green) VAMP4 expression (red) and a merged image. Arrows indicate example nerve terminals. Scale bar indicates 20 μm. **C)** Quantification of VAMP4 expression as a percentage of untransfected control (Scr, blue bars; VAMP4 KD, orange bars ± SEM (n = 4 coverslips for both, *** p<0.01, students t test). **D)** CGNs were transfected with synaptophysin-pHluorin (syp-pH) and either Scr or VAMP4 shRNA and stimulated with an action potential train (40 Hz 10 s). Bar indicates period of stimulation (n = 8 Scr, n = 6 VAMP4 KD, ns two-way-ANOVA). **E)** CGNs were transfected with either GFP, wild-type or L25A VAMP4-pHluorin and stimulated with 400 action potentials (40 Hz) in the presence of 50 μM TMR-dextran. Quantification of TMR-dextran puncta per μm ± SEM normalised to GFP control ± SEM (n = 23 GFP, n = 24 VAMP4 WT, n = 25 VAMP4 L25A, ns, one-way ANOVA).

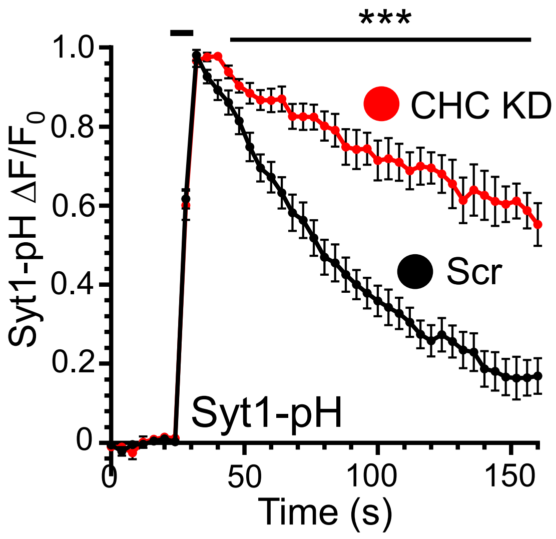

Supplementary Figure 8 – *Related to discussion section paragraph 2: Synaptotagmin-1-pHluorin retrieval is retarded by CHC shRNA.* Hippocampal neurons transfected with synaptotagmin-1-pHluorin (Syt1-pH) and either Scr or CHC KD shRNA were stimulated (40 Hz 5 s) as indicated by bar. Average time course $\Delta F/F_0$ ± SEM of the evoked syt1-pH response is displayed (n = 10 Scr, n = 9 CHC KD, ***=$p<0.001$, two-way ANOVA).
